# Efficient CO_2_ Activation Mediated by Triplet Pnictinidenes

**DOI:** 10.3390/molecules31183191

**Published:** 2026-09-10

**Authors:** Zheng-Feng Zhang, Ming-Der Su

**Affiliations:** 1 Department of Applied Chemistry, National Chiayi University, Chiayi 60004, Taiwan; 2 Department of Medicinal and Applied Chemistry, Kaohsiung Medical University, Kaohsiung 80708, Taiwan

**Keywords:** CO_2_, Group 15, triplet pnictinidenes, singlet–triplet crossing, intersystem crossing, spin-orbit coupling, minimum-energy crossing points, activation strain model

## Abstract

The present investigation delineates the mechanistic landscape governing the [1 + 2] cycloaddition of CO_2_ with triplet monomeric pnictinidenes (**[G15–Rea]^3^**; G15 = Group 15 element) and identifies the principal energetic factors controlling their periodic reactivity. The computed potential-energy profiles support a stepwise mechanism in which cycloaddition is initiated on the triplet potential-energy surface through **[G15–TS]^3^**, followed by a singlet–triplet surface-crossing event at **[G15–T/S]** and subsequent relaxation to the singlet heterocyclic products **[G15–Prod]^1^**. ASM analysis reveals that the activation barriers are governed primarily by the deformation penalty associated with CO_2_, whereas deformation of the pnictinidene and interfragment interaction contribute comparatively little to the overall barrier. The systematic increase in this deformation penalty with increasing G15 atomic radius provides a direct energetic basis for the progressive attenuation of reactivity from the lighter to the heavier pnictinidenes. Taken together, these findings establish a deformation-controlled origin for the periodic reactivity trend and provide a qualitative theoretical framework for assessing the feasibility and mechanistic implications of the proposed experimental observations.

## 1. Introduction

Carbon dioxide (CO_2_), predominantly generated from fossil fuel combustion, accumulates in the atmosphere and contributes significantly to global warming [1]. While a wide range of materials—including porous solids [2,3,4] and metal–organic frameworks (MOFs)—have been developed for CO_2_ capture and sequestration, these approaches largely emphasize storage over utilization [5,6,7]. In contrast, the catalytic conversion of CO_2_ into value-added C_1_ feedstocks has emerged as a compelling alternative [8,9,10,11]. In particular, Olah and co-workers established CO_2_-to-methanol conversion as a cornerstone of the “methanol economy,” providing a blueprint for a carbon-neutral energy cycle and stimulating extensive research into catalytic CO_2_ transformation [12,13,14,15,16,17].

The inherent thermodynamic stability and low reactivity of CO_2_, arising from its linear and nonpolar nature, present a fundamental challenge to its chemical conversion, often necessitating substantial energy input. Although CO_2_ can react with strong nucleophiles and coordinatively unsaturated transition-metal centers, its interaction with hydroxide ions typically yields inert bicarbonate species [18,19]. In contrast, main-group strategies for CO_2_ activation have only recently emerged (For instance: [20,21]). In this context, advances in main-group chemistry have propelled low-valent species to the forefront [22,23,24,25,26].

**Scheme 1:**
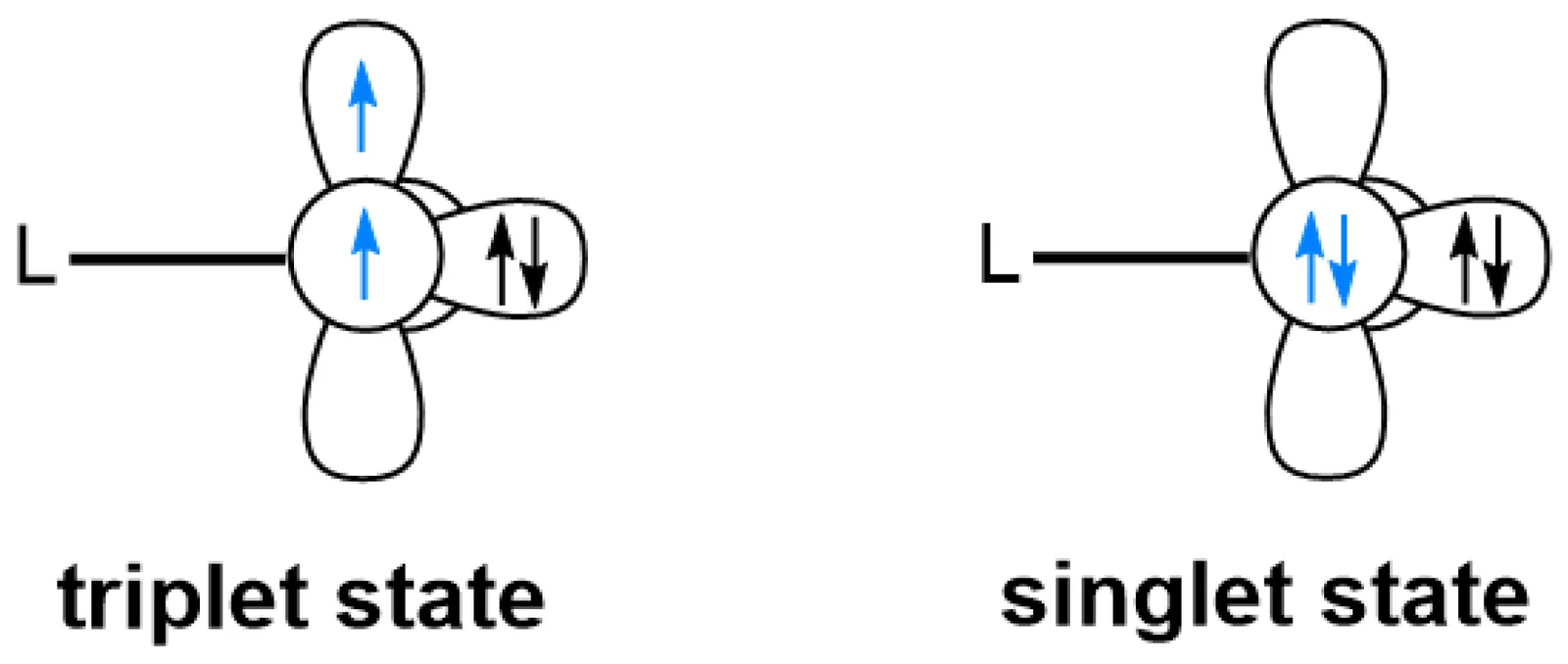
Schematic representation of the triplet and singlet electronic structures of pnictinidenes (**L–G15**). The electrons are color-coded to facilitate the distinction between the triplet and singlet states.

In a pioneering study, Holthausen, Schneider, and co-workers successfully synthesized and structurally characterized **L_1_–N** (L_1_ = Pt–N(CHCHP*t*Bu_2_)_2_), representing the first stable platinum metallonitrene [27]. Owing to its distinctive two-lone-pair valence electronic structure, this species can access either singlet or triplet electronic states depending on the supporting ligand environment [28]. The electronic structures depicted in Scheme 1 underscore the fundamental distinction between triplet and singlet pnictinidenes. Triplet species exhibit an open-shell configuration comprising one lone pair and two singly occupied mutually orthogonal p orbitals, whereas singlet species adopt a closed-shell electronic arrangement featuring two lone pairs and a vacant p orbital oriented perpendicular to the occupied orbital framework. Notably, experimental studies established **L_1_–N** as having a triplet ground state, making it a rare example of a neutral, monocoordinate nitrogen center that exhibits transition-metal-like reactivity [27]. This unusual electronic structure enables a wide range of intermolecular transformations, including phosphoraneiminato formation and facile nitrogen-atom insertion into C–H, B–H, and B–C bonds [27].

**Scheme 2:**
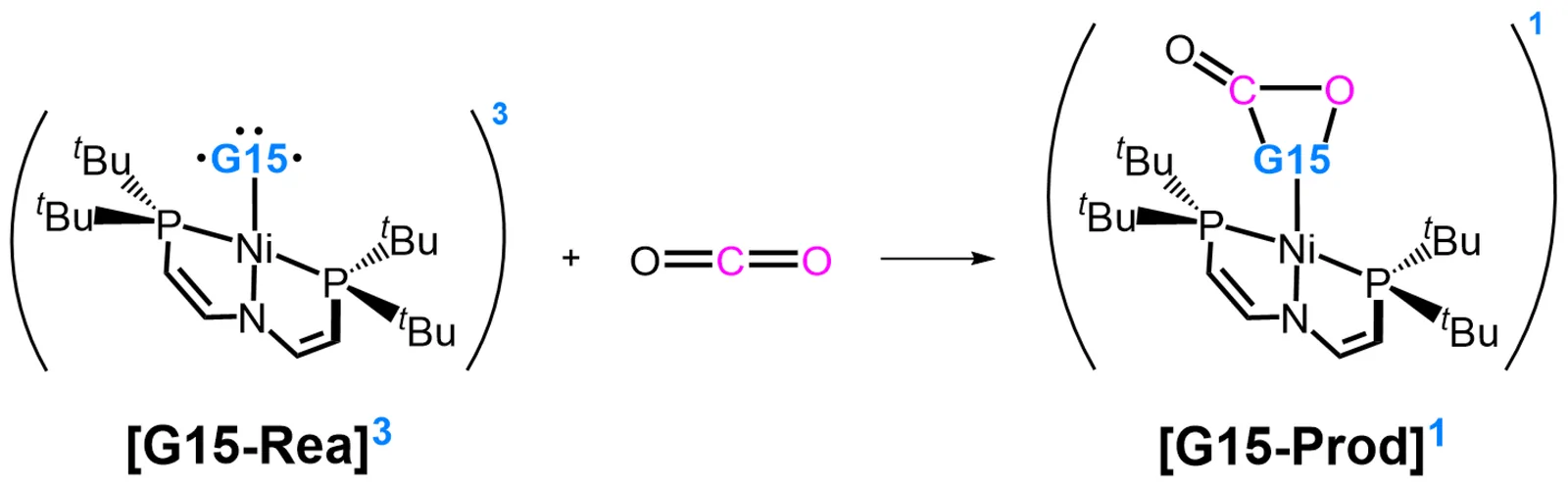
Reaction model for the [1 + 2] cycloaddition of CO_2_ with triplet pnictinidenes **L_2_–G15** (**[G15–Rea]^3^**; L_2_ = Ni–N(CHCHP*t*Bu_2_)_2_) possessing a neutral monocoordinate Group 15 center. Blue represents the spin state. Red represents the sites where the molecule participates in bonding.

The sustained development of synthetic methodologies has recently brought triplet pnictinidenes (**L–G15**, where G15 denotes a Group 15 element) within experimental reach. As a consequence, eleven structurally characterized examples have now been reported (Figure S1, Supporting Information) [27,29,30,31,32,33,34,35,36,37,38,39,40,41], providing unprecedented opportunities to explore the reactivity of these unusual open-shell main-group species. Despite their remarkable stability, these compounds retain substantial intrinsic reactivity. This raises an intriguing question: can triplet-ground-state pnictinidenes efficiently activate and capture the relatively inert CO_2_ molecule, and how does the nature of the Group 15 center influence the reactivity patterns and associated activation barriers of these processes? Because these fundamental mechanistic issues remain challenging to elucidate experimentally, quantum chemical calculations were employed to provide detailed insights into their electronic structures, bonding characteristics, and reaction mechanisms. Accordingly, the present study systematically investigates the CO_2_ capture reactions of triplet **L_2_–G15** species (**[G15–Rea]^3^**; L_2_ = Ni–N(CHCHP*t*Bu_2_)_2_) through a [1 + 2] cycloaddition pathway, as illustrated in Scheme 2.

This work aims to contribute to the mitigation of atmospheric CO_2_ accumulation through the development of strategies for its conversion into value-added chemicals. By elucidating the inherent triplet-state reactivity of pnictinidenes, the study reveals the key mechanistic determinants governing the interactions of L_2_–G15 species with CO_2_, thereby enabling improved predictive capabilities and rational molecular design. The computational results provide a comprehensive theoretical foundation for triplet Group 15 pnictinidene–CO_2_ chemistry.

It should be emphasized that a critical consideration in interpreting the present spin-state pathways is that the probability of intersystem crossing (ISC) cannot be established from the relative energetic ordering of the participating spin surfaces alone. Rather, spin-state conversion is governed by the interplay between spin–orbit coupling (SOC) and the energy separation of the interacting electronic states, together with the nonadiabatic nuclear dynamics in the vicinity of the crossing region. This effect is particularly consequential for heavy-element-containing species, for which enhanced SOC can facilitate the electronic-state mixing required for efficient spin conversion near a minimum-energy crossing point (MECP). Although the Landau–Zener formalism provides a theoretical framework for estimating ISC transition probabilities and associated rate constants, quantitatively predictive treatment would require an explicit and sufficiently rigorous description of the underlying nonadiabatic dynamics, which lies beyond the scope of the present computational methodology. Accordingly, the present results are interpreted primarily as establishing the energetic accessibility and mechanistic plausibility of the proposed pathways, together with the qualitative factors governing their activation barriers, rather than as providing quantitative ISC kinetic parameters.

## 2. Methodology

All molecular structures, including reactants, intermediates, transition states, and products in both singlet and triplet spin manifolds, were fully optimized using restricted and unrestricted density functional theory calculations at the M06-2X [42]-D3 [43,44,45]/def2-SVP [46] level without enforcing molecular symmetry. The Berny optimization algorithm [47] was employed for all geometry optimizations. Stationary points were rigorously characterized by analytical Hessian calculations [48] and vibrational frequency analyses, identifying minima by the absence of imaginary frequencies and transition states by the presence of a single imaginary frequency associated with the reaction coordinate. Intrinsic reaction coordinate (IRC) [49] calculations were subsequently performed to confirm that each transition state smoothly connects the corresponding reactant and product minima along the minimum-energy pathway. Thermal corrections and activation parameters were evaluated at 298.15 K within the harmonic oscillator and ideal-gas approximations using standard statistical thermodynamic methodologies [50].

The activation strain model (ASM) [51,52,53,54,55], alternatively known as the distortion/interaction model [56], was employed to quantitatively dissect the energetic origin of the activation barriers for the reactions of triplet pnictinidene (**L_2_–G15**) with CO_2_. Within the ASM framework, the activation energy (Δ*E*_ACT_) is decomposed into the deformation energies of the individual reacting fragments (∆*E*_DEF,[G15-Rea]_^3^ and ∆*E*_DEF,CO2_) and the interaction energy (Δ*E*_INT_) between the distorted fragments. The deformation energies represent the energetic penalty required to distort each fragment from its equilibrium geometry to the geometry adopted at a given point along the reaction coordinate, whereas the interaction energy reflects the stabilizing attractive interactions established between the distorted fragments. The interplay between these opposing energetic contributions ultimately determines the magnitude of the activation barrier, as summarized in Equation (1).

Δ*E*_ACT_ = ∆*E*_DEF,[G15-Rea]_^3^ + ∆*E*_DEF,CO2_ + Δ*E*_INT_ (1)

Energy decomposition analyses (EDAs) [57,58,59,60,61,62] were conducted using the ADF 2017.112 quantum chemical package [63]. All calculations were performed at the ZORA [64,65]-M06-2X-D3/TZ2P [66] level employing a triple-ζ Slater-type basis set augmented with two polarization functions. The basis functions consisted of uncontracted Slater-type orbitals, while the frozen-core approximation was applied to the chemically inactive core electrons. Scalar relativistic effects were explicitly incorporated through the zero-order regular approximation (ZORA) [64,65]. To ensure methodological consistency, all EDA calculations were carried out on molecular geometries optimized independently at the M06-2X-D3/def2-SVP level, corresponding to the composite computational protocol ZORA-M06-2X-D3/TZ2P//M06-2X-D3/def2-SVP.

To characterize the mechanism of intersystem crossing, the singlet–triplet crossing seam was explored by locating minimum-energy crossing points (MECPs). Since spin inversion constitutes an intrinsically nonadiabatic process [67], transitions between the two spin states become possible only when the corresponding potential energy surfaces approach energetic degeneracy, where spin–orbit coupling mediates population transfer. MECP optimizations were carried out using Harvey’s algorithm [68,69,70], and all optimized stationary points and crossing structures were obtained using Harvey’s code interfaced with GAUSSIAN 16 (Revision B.01) [71].

## 3. Results and Discussion

**Figure 1:**
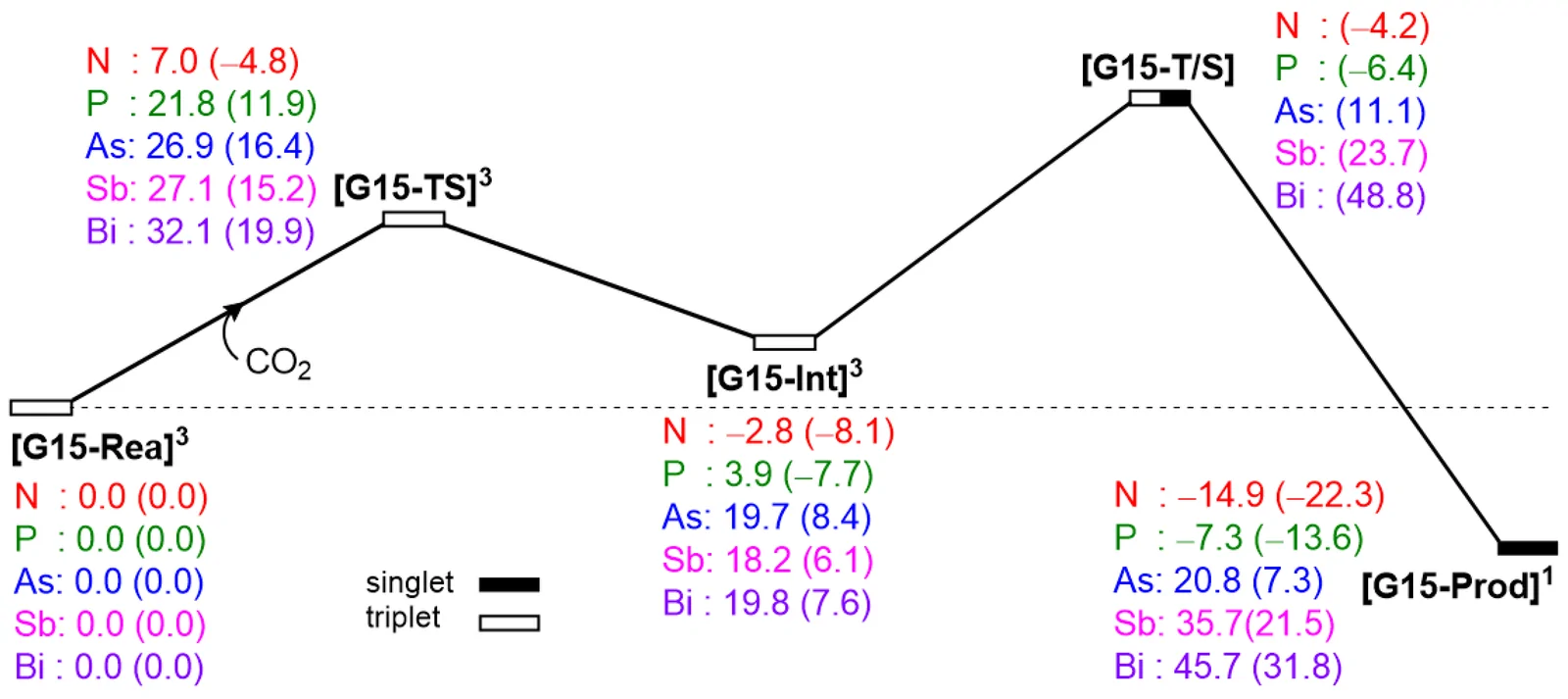
Calculated energy profiles for the [1 + 2] cycloaddition of CO_2_ with triplet pnictinidenes (**L_2_-G15**; L_2_ = Ni–N(CHCHPtBu_2_)_2_), obtained at the M06-2X-D3/def2-SVP level. Energies are referenced to the corresponding monomeric triplet **[G15-Rea]^3^** species. Gibbs free energies (Δ*G*) and electronic energies (Δ*E*; in parentheses) are reported in kcal mol^−1^. The optimized structures of the key stationary points along the triplet reaction pathways are provided in Figure 2. For clarity, the triplet pathways and singlet–triplet crossing points are depicted within a unified energy diagram. Minor deviations in the relative energy ordering for the As, Sb, and Bi systems may therefore be observed. The dashed line represents the energy value of the reactants.

**Figure 2:**
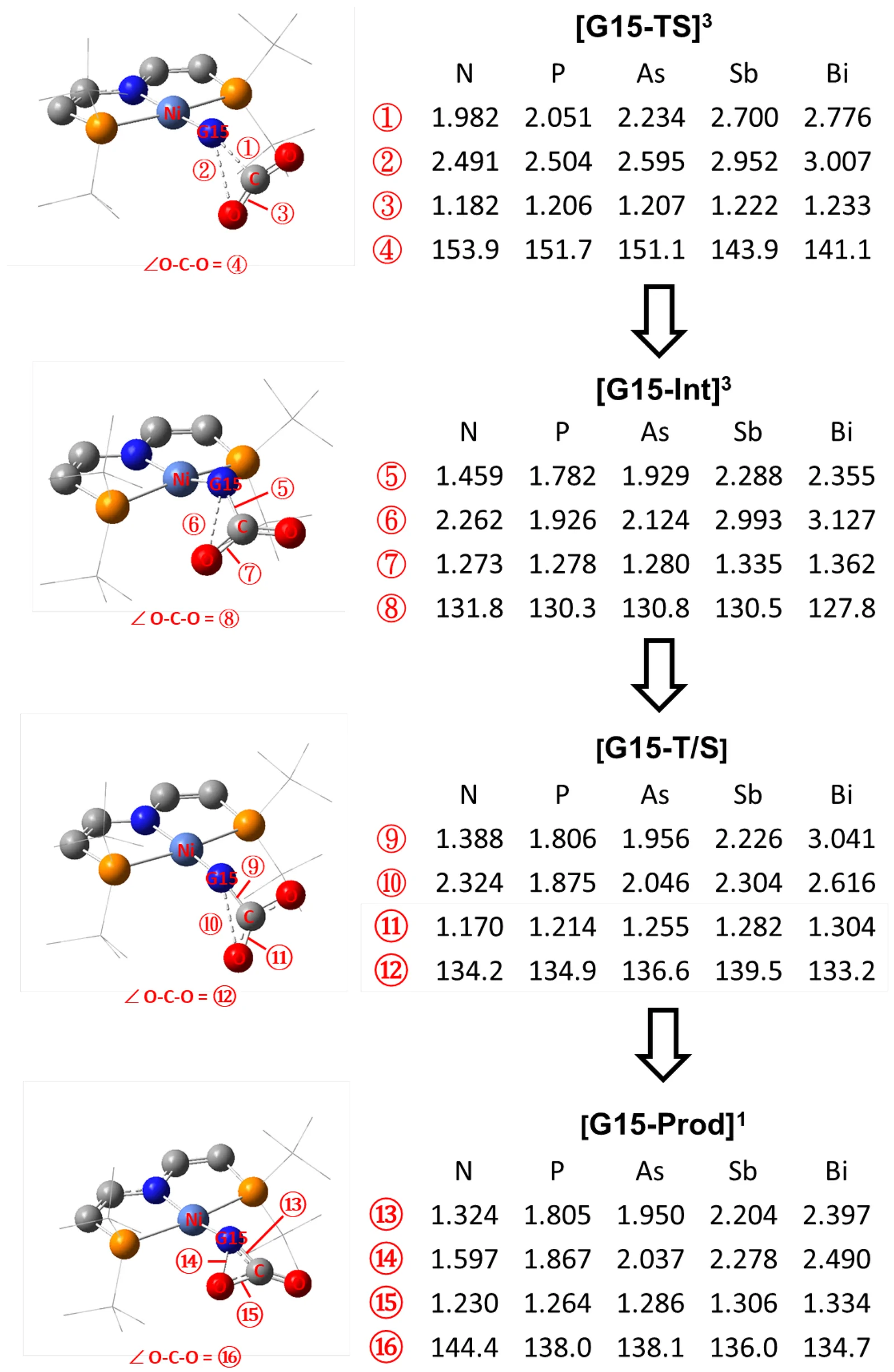
Geometrical parameters of the representative stationary points **[G15-TS]^3^**, **[G15-Int]^3^**, **[G15-T/S]**, and **[G15-Prod]^1^** on the potential energy surface for the [1 + 2] cycloaddition of CO_2_ mediated by triplet pnictinidene **[G15-Rea]^3^**. Structures were optimized at the M06-2X-D3/def2-SVP level of theory. Distances are reported in Å and angles in degrees. Relative energies are provided in Figure 1. Hydrogen atoms not participating in the reaction have been omitted.

Figure 1 presents the relative energies of key stationary points along the pnictinidene-mediated CO_2_ cycloaddition pathway shown in Scheme 2, computed at the M06-2X-D3/def2-SVP level of theory. All energies are referenced to the monomeric triplet pnictinidene (**[G15-Rea]^3^** or **(L_2_-G15)^3^**). Figure 2 shows the optimized structures of key stationary points along the [1 + 2] cycloaddition pathway of CO_2_ mediated by triplet pnictinidenes, including **[G15-TS]^3^**, **[G15-Int]^3^**, **[G15-T/S]**, and the singlet product **[G15-Prod]^1^**. For monomeric pnictinidenes **[G15-Rea]** (**L_2_-G15**; Figures S2 and S3), the singlet–triplet energy gap Δ*E*_st_ (=*E*_triplet_ − *E*_singlet_) follows a well-defined periodic trend across Group 15: N (−16.4) > P (−18.2) > As (−22.5) > Sb (−27.6) > Bi (−34.4). This trend indicates a systematic stabilization of the triplet state (**[G15-Rea]^3^**) relative to the singlet state (**[G15-Rea]^1^**), with |Δ*E*_st_| increasing down the group.

Two mechanistically distinct pathways were considered for the activation of singlet-state CO_2_ by triplet-ground-state pnictinidenes (**[G15-Rea]^3^**). The corresponding reaction sequences are:

Path I: **[G15-Rea]^3^** + **[CO_2_]^1^** → **[G15-Rea]^1^** + **[CO_2_]^1^** → **[G15-TS]^1^** → **[G15-Prod]^1^**

Path II: **[G15-Rea]^3^** + **[CO_2_]^1^** → **[G15-TS]^3^** *→* **[G15-Int]^3^** *→* **[G15-T/S]** *→* **[G15-Prod]^1^**

We initially investigated Path I, which entails CO_2_ capture by singlet **[G15-Rea]^1^** through singlet **[G15-TS]^1^**, followed by formation of singlet **[G15-Prod]^1^**. The principal impediment to this pathway originates from the intrinsic spin-state energetics of the reactive pnictinidene. DFT calculations demonstrate that the triplet state of **[G15-Rea]^3^** is substantially stabilized, lying 16.4–34.4 kcal/mol below its singlet counterpart. This energetic preference becomes progressively more pronounced on descending Group 15, as evidenced by the systematic growth of |Δ*E*_st_|. Consequently, the singlet reaction surface becomes increasingly inaccessible across the series, imposing an intrinsic energetic penalty before the system can engage CO_2_ along Path I. This unfavorable spin-state energetics is expected to propagate into the reaction coordinate, resulting in a substantially elevated barrier for singlet-state CO_2_ activation. Path I is therefore intrinsically disfavored and can be excluded from the mechanistic landscape considered herein.

The mechanistic landscape of Path II was systematically mapped by characterizing the relevant potential-energy surfaces for the [1 + 2] cycloaddition of triplet-ground-state pnictinidenes (**[G15-Rea]^3^**) with singlet-state CO_2_ at the M06-2X-D3/def2-SVP level of theory. As depicted in Figure 1, the reaction coordinate is characterized by a direct approach of **[G15-Rea]^3^** toward CO_2_, followed by formation of the triplet transition state **[G15-TS]^3^**, with no intervening pre-reactive minimum identified at this level of theory. With **[G15-Rea]^3^ +** CO_2_ taken as the reference state, the activation free energies exhibit a systematic increase across the Group 15 series: **[N-TS]^3^** (7.0) < **[P-TS]^3^** (21.8) < **[As-TS]^3^** (26.9) < **[Sb-TS]^3^** (27.1) < **[Bi-TS]^3^** (32.1). This pronounced trend establishes the Group 15 identity as a key determinant of the activation energetics.

The reaction mechanism predicted by the present calculations involves initial traversal of the triplet transition state **[G15-TS]^3^**, followed by the formation of the triplet intermediate **[G15-Int]^3^**. The relative Gibbs free energies (Δ*G*_INT_, kcal/mol) of the intermediates exhibit the trend **[N-Int]^3^** (−2.8) < **[P-Int]^3^** (3.9) < **[Sb-Int]^3^** (18.2) < **[As-Int]^3^** (19.7) < **[Bi-Int]^3^** (19.8). The conversion of **[G15-Int]^3^** to the crossing structure **[G15-T/S]** is kinetically accessible for the N, P, As, and Sb congeners, with estimated activation barriers of 2.7–17.6 kcal/mol. However, the analogous process for **[Bi-Int]^3^** is associated with a significantly elevated barrier of 41.2 kcal/mol.

Subsequent to the generation of the triplet intermediate **[G15-Int]^3^**, the system undergoes an intersystem crossing (ISC) process at **[G15-T/S]**, thereby producing the singlet three-membered heterocyclic species **[G15-Prod]^1^**. A clear periodic trend is observed for the singlet–triplet crossing point energies (Δ*E*_T/S_, kcal/mol), which increase in the sequence **[N–T/S]** (−6.4) < **[P–T/S]** (−4.2) < **[As–T/S]** (11.1) < **[Sb–T/S]** (23.7) < **[Bi–T/S]** (48.8). The rising energetic demand for reaching the crossing point could imply a gradual reduction in the propensity for spin crossover with increasing atomic weight of the pnictogen center. (It should be emphasized that, as noted in the Introduction, MECP energies alone are insufficient to determine the efficiency of ISC. Accordingly, our previous statement that the “propensity for spin crossover” decreases down the group is not sufficiently justified in the absence of explicit SOC calculations. We therefore restrict our conclusion to the decreasing energetic accessibility of the singlet–triplet crossing region and refrain from implying that spin-state conversion necessarily occurs at the MECP.) Likewise, the M06-2X-D3-calculated Gibbs free reaction energies (Δ*G*_RXN_, kcal mol^−1^) show a systematic increase across the series: **[N-Prod]^1^** (−14.9) < **[P-Prod]^1^** (−6.9) < **[As-Prod]^1^** (20.8) < **[Sb-Prod]^1^** (35.7) < **[Bi-Prod]^1^** (45.7). This trend suggests that stabilization of the products diminishes markedly with increasing atomic size and relativistic character of the pnictogen center, ultimately suppressing product formation for heavier congeners. Taken together, the kinetic and thermodynamic profiles establish a clear reactivity boundary within Group 15 pnictinidenes. Only the lighter systems, **[N-Rea]^3^** and **[P-Rea]^3^**, are capable of undergoing facile [1 + 2] cycloaddition with CO_2_ to yield three-membered heterocycles, whereas the heavier congeners (**[As-Rea]^3^**, **[Sb-Rea]^3^**, and **[Bi-Rea]^3^**) are associated with endergonic reaction landscapes that effectively preclude productive cycloaddition. This trend highlights a pronounced attenuation of reactivity with increasing pnictogen atomic number.

**Figure 3:**
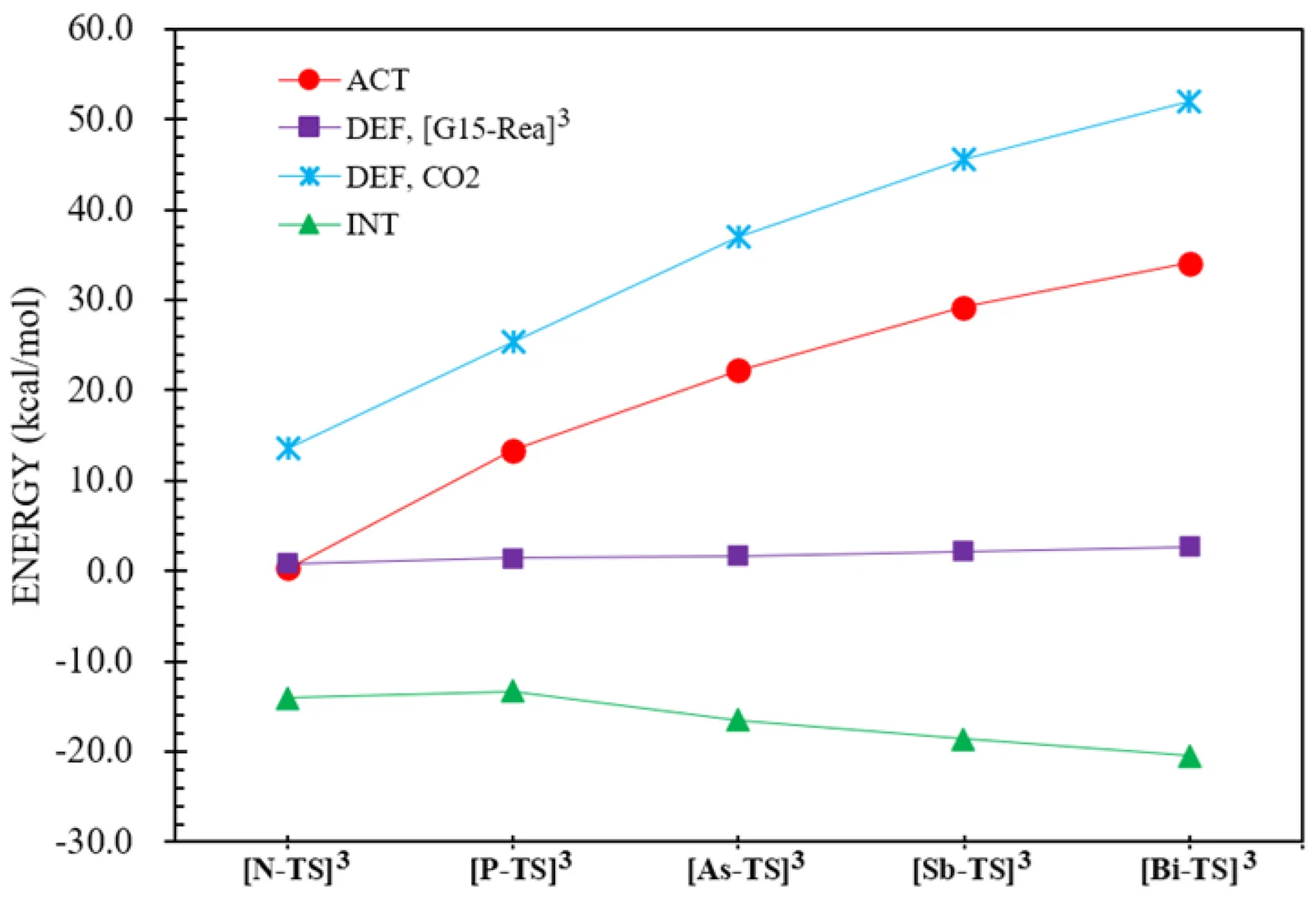
Activation strain analysis of **[G15-TS]^3^**, with all numerical results corresponding to the values reported in Table 1 for consistency and reference.

To elucidate the physical origin of the activation barrier, activation strain model (ASM) analysis was performed on **[G15-TS]^3^** by partitioning the system into **[G15-Rea]^3^** and CO_2_ fragments. Within this energy decomposition framework, the activation energy (Δ*E*_ACT_) is separated into strain (∆*E*_DEF_) and interaction (Δ*E*_INT_) components, enabling a quantitative assessment of the factors governing the reaction barrier. The strain energy (∆*E*_DEF_), defined as the sum of fragment deformation energies ∆*E*_DEF,[G15-Rea]_^3^ and ∆*E*_DEF,CO2_, accounts for the structural distortion of the reacting species, whereas Δ*E*_INT_ describes their mutual electronic interaction. As revealed in Table 1 and Figure 3, both ∆*E*_DEF,[G15-Rea]_^3^ and Δ*E*_INT_ remain largely invariant across the Group 15 series, whereas ∆*E*_DEF,CO2_ exhibits a pronounced increase that closely mirrors the trend in Δ*E*_ACT_, clearly identifying CO_2_ deformation as the dominant factor governing the activation barrier.

The observed trend can be rationalized by two principal factors. First, the substantially larger size and greater structural rigidity of triplet **[G15–Rea]^3^** relative to CO_2_ result in negligible deformation of this fragment upon approach. In contrast, the CO_2_ moiety undergoes pronounced geometric distortion to optimize orbital overlap with the G15 center, thereby contributing predominantly to ∆*E*_DEF,CO2_ rather than ∆*E*_DEF,[G15-Rea]_^3^ or Δ*E*_INT_ (Figure 3), and ultimately governing the overall strain energy profile. Second, a pronounced periodic effect arises from the substantial increase in atomic radius down Group 15, from 71 pm for N to 148 pm for Bi [72]. Consequently, as CO_2_ approaches the pnictinidene center, progressively greater activation of the CO_2_ framework—primarily manifested as one C–O bond elongation—is required to achieve optimal orbital overlap with the increasingly diffuse valence orbitals of the heavier pnictogen atoms. This enhanced distortion directly contributes to the increase in ∆*E*_DEF,CO2_ observed across the series. In addition, the developing interaction leads to a marked geometric reorganization of CO_2_ from a linear to a bent configuration. This distortion, originating from steric crowding between the oxygen atom and the L_2_ substituent, contributes significantly to the increase in ∆*E*_DEF,CO2_. Overall, the activation barrier trend can be rationalized by the combined effects of increasing pnictogen atomic size and the associated deformation requirements of the CO_2_ fragment. As the Group 15 element becomes heavier, effective orbital overlap with the increasingly diffuse valence orbitals of the pnictogen center demands progressively greater C–O bond elongation and O–C–O angle bending. These structural distortions impose a substantial energetic penalty on the CO_2_ fragment, leading to a pronounced increase in ∆*E*_DEF,CO2_. Consequently, CO_2_ deformation emerges as the dominant factor controlling the activation barrier and the overall reactivity pattern of the [1 + 2] cycloaddition of CO_2_ with monomeric triplet pnictinidenes **[G15–Rea]^3^**.

The optimized triplet transition-state structures (**[G15–Rea]^3^**) shown in Figure 1 provide direct structural evidence supporting this interpretation. As the atomic radius of the Group 15 center increases, the bent CO_2_ fragment exhibits progressively greater C–O bond elongation, following the order **[N–TS]^3^** (1.5%) < **[P–TS]^3^** (3.5%) < **[As–TS]^3^** (3.6%) < **[Sb–TS]^3^** (4.9%) < **[Bi–TS]^3^** (5.8%), relative to the equilibrium C–O bond length of free CO_2_ (1.156 Å). Furthermore, the extent of O–C–O angle bending closely parallels the activation barrier trend, with deviations from the linear geometry of CO_2_ (180.0°) increasing systematically along the series: **[N–TS]^3^** (14.5%) < **[P–TS]^3^** (15.7%) < **[As–TS]^3^** (16.1%) < **[Sb–TS]^3^** (20.1%) < **[Bi–TS]^3^** (21.6%). This progressive geometric distortion reflects a gradual evolution of the transition-state character from reactant-like to product-like across the Group 15 series. In particular, **[N-TS]^3^** corresponds to an early transition state with minimal CO_2_ deformation, whereas **[Bi-TS]^3^** represents a substantially later transition state characterized by extensive structural reorganization. According to Hammond’s postulate [73], such a shift toward increasingly product-like transition states is expected to accompany higher activation barriers. This prediction is fully consistent with the computed DFT energy profiles presented in Figure 1.

It should be stressed that the observed increase in CO_2_ deformation energy does not necessarily establish deformation as the fundamental origin of the barrier trend. Because the transition states become progressively later from N to Bi, the enhanced deformation may partly represent a consequence of the changing transition-state geometry. We therefore interpret the deformation–barrier relationship as a correlation and deliberately avoid assigning it a uniquely dominant causal role. Furthermore, ISC efficiency cannot be inferred solely from the relative energies of the MECPs, as the crossing probability is influenced by additional dynamical and electronic factors. In view of these limitations, we restrict the present mechanistic discussion to Path I and Path II, which provide the most plausible descriptions of the reaction. Effects associated with molecular reorganization and nonadiabatic spin crossing remain beyond the present scope but may be considered in future investigations. The Landau–Zener formalism provides one possible framework for such a quantitative treatment, particularly for estimating ISC transition probabilities and the corresponding rate constants.

## 4. Conclusions

The persistent accumulation of anthropogenic CO_2_ in the atmosphere necessitates the development of efficient and environmentally benign strategies for its capture and utilization. Molecular approaches based on non-toxic main-group compounds are particularly attractive because they offer opportunities for controlling CO_2_ reactivity through electronic-structure design. Whereas the capture of CO_2_ by singlet-state main-group species has been extensively studied, the reactivity of triplet-ground-state main-group molecules toward singlet CO_2_ remains an essentially unexplored frontier. Herein, we report a systematic theoretical investigation of this distinctive spin-state-dependent reactivity. To the best of our knowledge, this study constitutes the first theoretical exploration of CO_2_ capture by triplet-ground-state main-group molecules and establishes mechanistic principles that may serve as a predictive foundation for experimental investigations of these unconventional CO_2_-capture reactions. The mechanistic insights obtained herein provide a fundamental understanding of this transformation and lead to the following key conclusions:

(1) Two distinct mechanistic pathways were considered for the reaction of singlet-state CO_2_ with triplet-ground-state pnictinidenes. Comparative analysis of the computed energy landscapes establishes Path II as the energetically preferred reaction channel. The pathway is initiated by [1 + 2] cycloaddition on the triplet potential-energy surface, with the reactants evolving through the triplet transition state **[G15-TS]^3^** and subsequently reaching the triplet intermediate **[G15–Int]^3^**. From this intermediate, the reaction proceeds toward the **[G15-T/S]** crossing seam, where spin-state conversion occurs and the system accesses the singlet potential-energy surface, ultimately yielding **[G15-Prod]^1^**. The complete mechanistic sequence can therefore be represented as:

**[G15-Rea]^3^** + CO_2_ *→*
**[G15-TS]^3^** *→*
**[G15-Int]^3^** *→*
**[G15-T/S]**
*→*
**[G15-Prod]^1^**

**Scheme 3:**
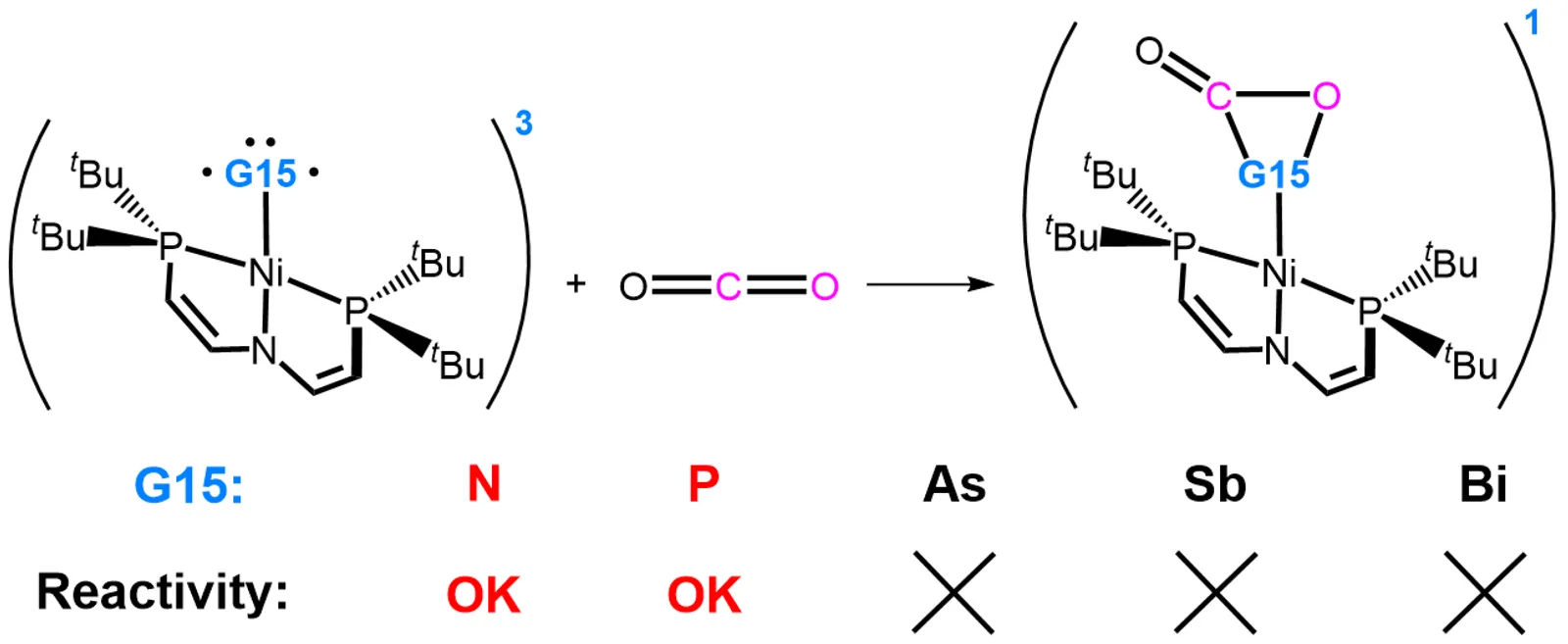
DFT-predicted [1 + 2] cycloaddition of CO_2_ mediated by triplet pnictinidenes **[G15-Rea]^3^**.

(2) The M06-2X-D3 calculations reveal a distinct periodic reactivity trend across the Group 15 series. The lighter triplet pnictinidenes **[N-Rea]^3^** and **[P-Rea]^3^** are predicted to undergo facile thermal [1 + 2] cycloaddition with CO_2_, ultimately yielding singlet three-membered heterocyclic products. In contrast, the corresponding heavier congeners **[As-Rea]^3^**, **[Sb-Rea]^3^**, and **[Bi-Rea]^3^** exhibit progressively less favorable reaction energetics and proceed through endergonic pathways, thereby rendering product formation thermodynamically unfavorable (Scheme 3).

(3) ASM results demonstrate that the CO_2_ fragment is the primary source of the activation barrier in the triplet **[G15-Rea]^3^**-mediated cycloaddition, and that its deformation energy increases progressively with pnictogen atomic size.
(4) The M06-2X-D3 study confirms the applicability of Hammond’s postulate, whereby earlier, more reactant-like transition states in the [1 + 2] cycloaddition of CO_2_ with triplet pnictinidenes are associated with reduced activation energies.

Overall, the mechanistic and electronic-structure insights uncovered in this study are expected to provide a robust foundation for the future development of triplet Group 15 pnictinidene chemistry, particularly in the context of CO_2_ capture, activation, and valorization, thereby stimulating further synergistic computational and experimental investigations in this rapidly evolving area.

## Figures and Tables

**Table 1:** The activation strain model (ASM) analysis of **[G15-TS]^3^** was employed to dissect the underlying energetic contributions, using the ZORA-M06-2X-D3/TZ2P//M06-2X-D3/def2-SVP level of theory ^(a)^.

Entry	[N-TS]^3^	[P-TS]^3^	[As-TS]^3^	[Sb-TS]^3^	[Bi-TS]^3^
Δ*E*_ACT_^(b)^	0.3	13.4	22.2	29.2	34.1
Δ*E*_DEF,[G15-Rea]_^3^	0.8	1.4	1.7	2.2	2.7
∆*E*_DEF,CO2_	13.6	25.3	37.0	45.6	51.9
Δ*E*_INT_	−14.1	−13.3	−16.5	−18.6	−20.5

^(a)^ All in kcal mol^−1^. ^(b)^ Δ*E*_ACT_ = ∆*E*_DEF,[G15-Rea]_^3^ + ∆*E*_DEF,CO2_ + Δ*E*_INT_.

## Data Availability

The original contributions presented in this study are included in the article/Supplementary Materials. Further in-quiries can be directed to the corresponding author.

## Supplementary Materials

The following supporting information can be downloaded at: https://www.mdpi.com/article/10.3390/molecules31183191/s1. References [27,29,30,31,32,33,34,35,36,37,38,39] are cited in the Supplementary Materials.
