# Supplementary material for: Efficient CO2 Activation Mediated by Triplet Pnictinidenes
Source: Molecules. 2026 Sep 10;31(18):3191. doi: 10.3390/molecules31183191 (PMC13609287; doi:10.3390/molecules31183191)
Supplement: Supplementary file 1 [file molecules-31-03191-s001.zip › molecules-4505243-supplementary.pdf]

Supporting Information

Efficient CO_2 Activation Mediated by Triplet Pnictinidenes

Zheng-Feng Zhang [1] and Ming-Der Su [1,2,*]

1 Department of Applied Chemistry, National Chiayi University, Chiayi 60004, Taiwan

2 Department of Medicinal and Applied Chemistry, Kaohsiung Medical University, Kaohsiung 80708, Taiwan

* Correspondence: midesu@mail.ncyu.edu.tw

==

Content:

Methodology--S3-S7

Table SA. Total number of atoms and basis functions ---------------------------------- S3

Table SB. The computed energies for the intersystem crossing points --------------- S3

Figure S1. Ten experimentally reported triplet pnictinidenes ---------------------S4-S5

Figure S2. Optimized structures for triplet pnictinidenes -------------------------------S6

Figure S3. Optimized structures for singlet pnictinidenes ------------------------------S7

Tables S1-S31 Cartesian coordinates of the stationary points---------------------S8-S98

Table SA. The total number of atoms and basis functions (M06-2X-D3/def2-SVP) involved in these molecular systems is summarized in the table. As shown therein, for instance, the **[Bi–T/S]** system comprises 68 atoms and 635 basis functions, representing a computationally demanding system. Even at the def2-SVP level, the calculations are already computationally intensive, and the use of larger basis sets, such as def2-TZVP, would result in a prohibitive increase in computational cost.

============================

CO_2 pathway (68 atom numbers):

N: 617 basis functions
P: 621 basis functions
As: 629 basis functions
Sb: 635 basis functions
Bi: 635 basis functions

============================

Table SB. The computed energies for the intersystem crossing points, denoted as **[G15-T/S]**.

Crossing Points	Triplet state (Hartree)	Singlet state (Hartree)
**[N-T/S]**	-3273.47231199	-3273.47234234
**[P-T/S]**	-3560.08316834	-3560.08318803
**[As-T/S]**	-5454.47414955	-5454.47416998
**[Sb-T/S]**	-3459.02861311	-3459.02863787
**[Bi-T/S]**	-3433.36652793	-3433.36653041

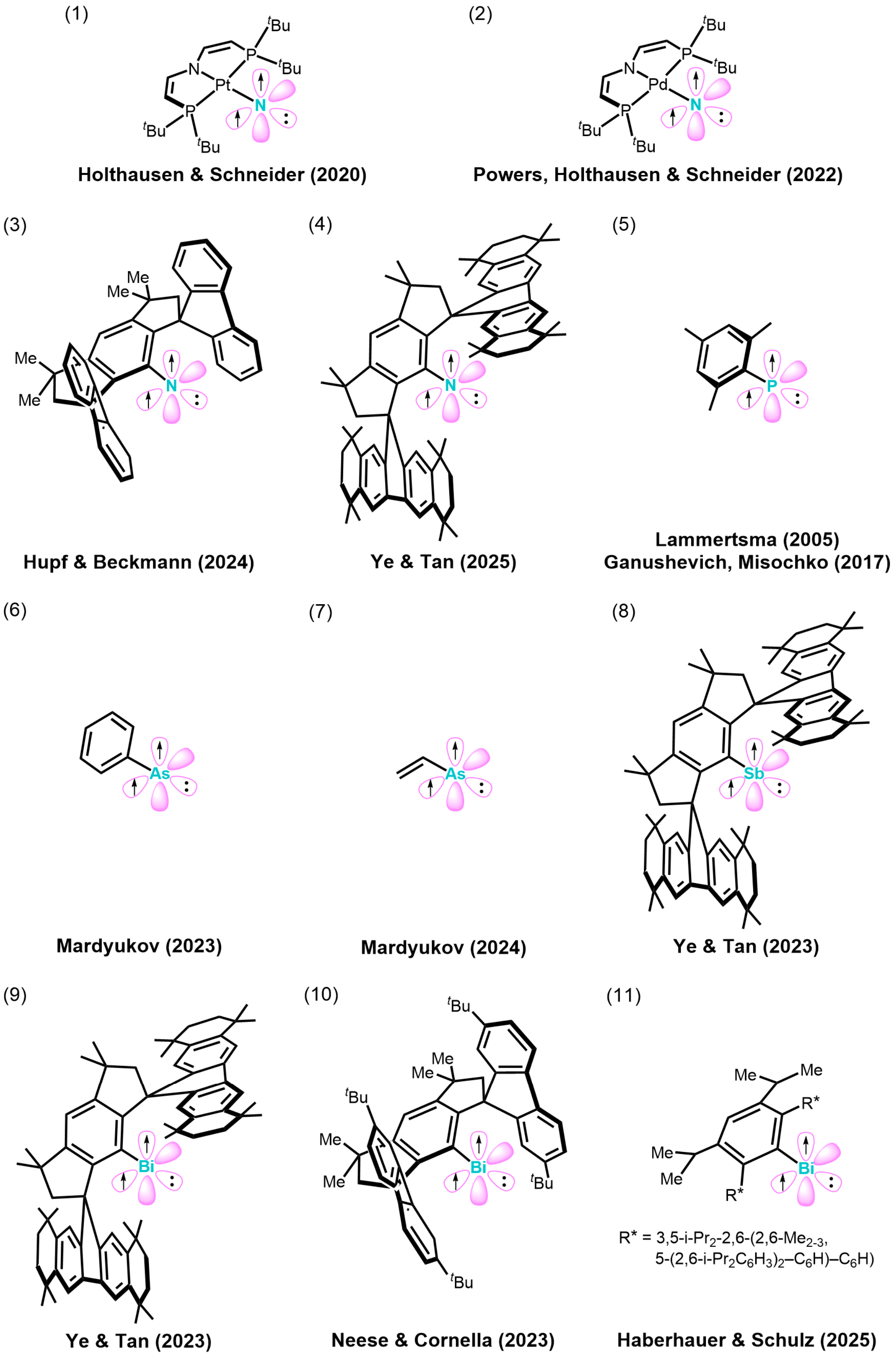

Figure S1. Eleven stable triplet pnictinidene (L–G15; G15 = N, P, As, Sb, and Bi) species experimentally reported to date (1–11).

References:

(1) J. Sun, J. Abbenseth, H. Verplancke, M. Diefenbach, B. de Bruin, D. Hunger, C. Würtele, J. van Slageren, M. C. Holthausen and S. Schneider, *Nat. Chem.*, 2020, **12**, 1054-1059.

(2) T. Schmidt-Räntsch, H. Verplancke, J. N. Lienert, S. Demeshko, M. Otte, G. P. Van Trieste III, K. A. Reid, J. H. Reibenspies, D. C. Powers, M. C. Holthausen and S. Schneider, *Angew. Chem. Int. Ed.*, 2022, **61**, e202115626.

(3) M. Janssen, T. Frederichs, M. Olaru, E. Lork, E. Hupf and J. Beckmann, *Science*, 2024, **385**, 318-321.

(4) D. Wang, W. Chen, H. Chen, Y. Chen, S. Ye and G. Tan, *Nat. Chem.*, 2025, **17**, 38-43.

(5) G. Bucher, M. L. G. Borst, A. W. Ehlers, K. Lammertsma, S. Ceola, M. Huber, D. Grote and W. Sander, *Angew. Chem. Int. Ed.*, 2005, **44**, 3289-3293.

(6) A. V. Akimov, Y. S. Ganushevich, D. V. Korchagin, V. A. Miluykov and E. Y. Misochko, *Angew. Chem. Int. Ed.*, 2017, **56**, 7944-7947.

(7) W. Qian, P. R. Schreiner and A. Mardyukov, *J. Am. Chem. Soc.*, 2023, **145**, 12755-12759.

(8) W. Qian, P. R. Schreiner and A. Mardyukov, *J. Am. Chem. Soc.*, 2024, **146**, 930-935.

(9) M. Wu, H. Li, W. Chen, D. Wang, Y. He, L. Xu, S. Ye and G. Tan, *Chem.*, 2023, **9**, 2573-2584.

(10) M. Wu, W. Chen, D. Wang, Y. Chen, S. Ye and G. Tan, *Nat. Sci. Rev.*, 2023, **10**, 169-175.

(11) Y. Pang, N. Nöthling, M. Leutzsch, L. Kang, E. Bill, M. van Gastel, E. Reijerse, R. Goddard, L. Wagner, D. SantaLucia, S. DeBeer, F. Neese and J. Cornella, *Science*, 2023, **380**, 1043-1048.

(12) Y. Schulte, T. Freese, C. Wölper, J. Schulte, G. Haberhauer and S. Schulz, *Angew. Chem. Int. Ed.*, 2025, **64**, e202508250.

Figure S2. Optimized structures at the M06-2X-D3/def2-SVP level (bond lengths in Å) for the triplet ground-state pnictinidene **[G15-Rea]**^{3} and singlet CO_2.

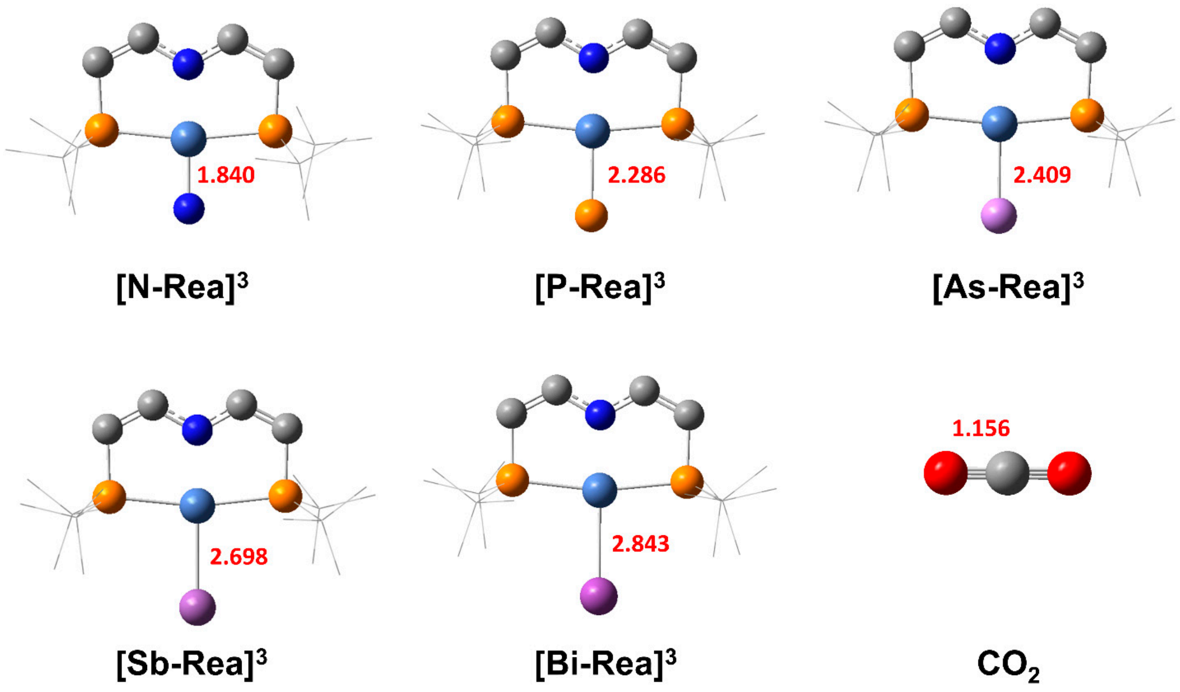

Figure S3. Optimized structures at the M06-2X-D3/def2-SVP level (bond lengths in Å) for the singlet pnictinidene **[G15-Rea]¹**.

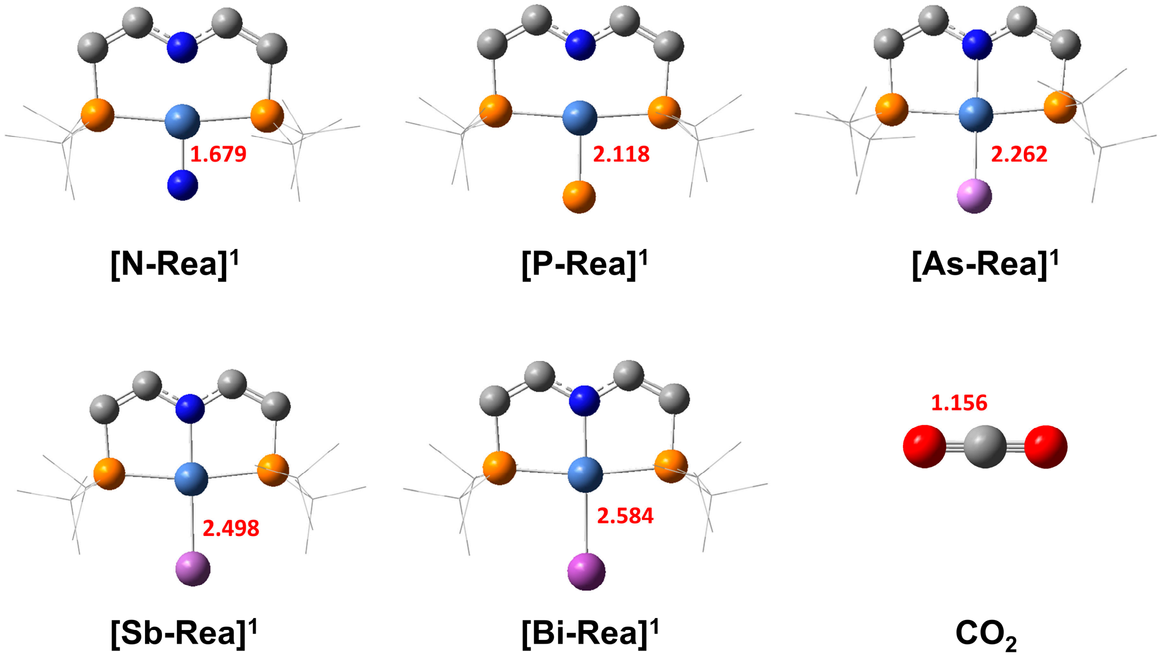

Figure S4. Single-point energies for the CO_2 [1 + 2] cycloaddition reactions of **L_2–G15** (L_2 = Ni–N(CHCHPtBu$_2$)$_2$), evaluated at the ωB97XD/def2-TZVP level using gas-phase geometries optimized at the M06-2X-D3/def2-SVP level (ωB97XD/def2-TZVP//M06-2X-D3/def2-SVP). Gibbs free energies (ΔG), electronic energies (ΔE; in parentheses), and single-point energies (in square brackets) are reported. Minor variations in the relative energetic ordering of selected stationary points are observed, particularly for the N, P, and Bi systems. Notably, the overall reactivity trend across the **L_2–G15** series remains essentially invariant upon increasing both the level of theory and basis-set size. Although the present ωB97XD/def2-TZVP single-point calculations provide useful additional support for the general trend of greater reactivity among the lighter elements relative to their heavier congeners, the detailed N→Bi ordering is not fully preserved, and the MECP energetics were not independently benchmarked at this level of theory. We therefore temper our claims regarding the robustness of the periodic reactivity trend and the mechanistic interpretation of ISC accordingly.

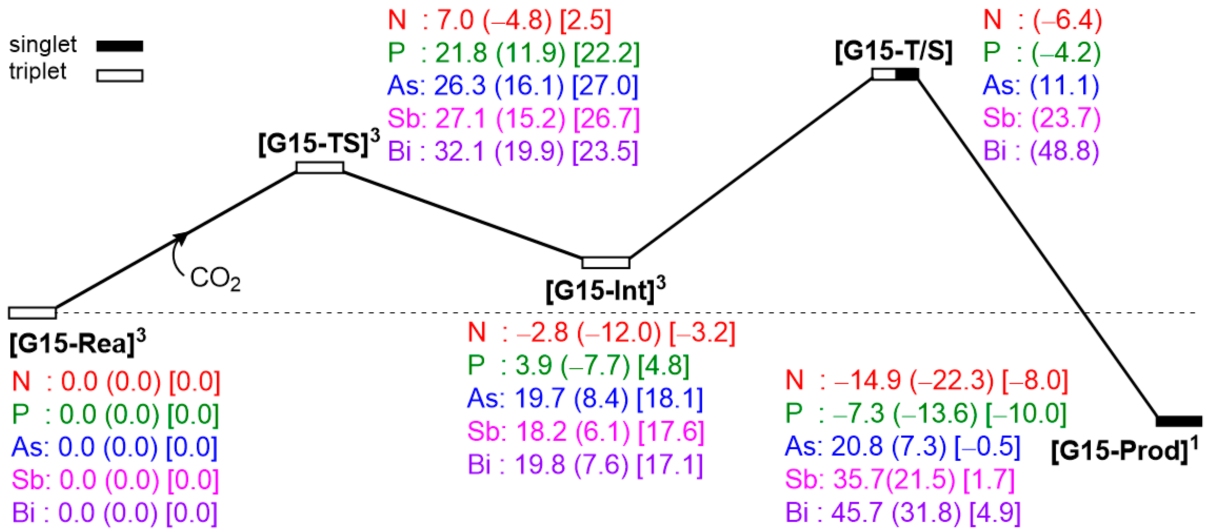

(All were calculated at the M06-2X-D3/def2-SVP level of theory)

Table S1

[N-Rea][3]

E : -3085.09525321 Hartree

G : -3084.587616 Hartree

Atomic Number	Coordinates (Angstroms) X	Y	Z
7	-2.546272	0.220803	-0.251391
28	-0.978417	-0.752892	0.458977
6	-3.616525	-0.480529	-0.710354
6	-2.535372	1.579973	-0.272327
6	-3.675769	-1.843066	-0.684555
15	-2.240565	-2.633869	0.053496
15	-0.066800	1.345108	0.710233
6	-1.472745	2.318972	0.158161
1	-4.459721	0.097346	-1.119131
1	-4.555026	-2.369891	-1.057372
1	-3.435735	2.085197	-0.654546
1	-1.500007	3.408678	0.120660
6	1.387976	1.770086	-0.401032
6	-1.476280	-3.724791	-1.270883
7	0.480711	-1.660017	1.119986
6	2.503326	0.733331	-0.209047
6	1.923670	3.186382	-0.186166

6	0.847506	1.637043	-1.832792
1	2.408783	3.294685	0.794567
1	1.125155	3.939840	-0.267468
1	2.679489	3.413479	-0.955864
1	0.388055	0.648839	-1.995740
1	1.681128	1.748033	-2.544119
1	0.091199	2.402129	-2.054289
1	2.983247	0.809672	0.774488
1	3.279901	0.897044	-0.973766
1	2.119516	-0.292658	-0.312390
6	-0.432393	-4.661937	-0.657005
6	-0.760839	-2.736139	-2.205737
6	-2.519163	-4.508993	-2.070159
1	0.262969	-4.108717	-0.005547
1	0.151945	-5.129351	-1.466095
1	-0.898858	-5.471413	-0.077094
1	-3.088512	-5.207206	-1.441927
1	-2.011854	-5.097899	-2.851814
1	-3.227683	-3.829991	-2.566224
1	0.077455	-2.247471	-1.684606
1	-1.449874	-1.958972	-2.572834
1	-0.357249	-3.278007	-3.076202
6	-2.832021	-3.590249	1.559386
6	-3.680260	-2.589319	2.358552
6	-1.627116	-3.994792	2.419971
6	-3.680145	-4.813183	1.207465
1	-0.980560	-3.131208	2.636635

1	-1.006121	-4.761214	1.939919
1	-1.991756	-4.409142	3.373922
1	-3.117486	-1.664656	2.564676
1	-3.958338	-3.042613	3.323136
1	-4.597879	-2.313962	1.821346
1	-4.521389	-4.548170	0.548664
1	-4.099526	-5.246847	2.130115
1	-3.083724	-5.595558	0.716462
6	0.221086	1.777121	2.515466
6	-0.903766	1.041471	3.261692
6	0.110982	3.277981	2.792640
6	1.567748	1.225099	2.991607
1	1.685855	0.167117	2.707457
1	1.616659	1.293892	4.090440
1	2.412976	1.800785	2.587418
1	-1.894572	1.311006	2.862752
1	-0.876631	1.312568	4.329388
1	-0.774661	-0.049277	3.179564
1	-0.880674	3.659066	2.508858
1	0.873354	3.859176	2.256584
1	0.247171	3.460275	3.871232

Table S2

[P-Rea]^3

E : -3371.70250842 Hartree

G : -3371.196140 Hartree

Atomic Number	Coordinates (Angstroms) X	Y	Z
7	-2.496187	0.190765	-0.230831
28	-0.928147	-0.782881	0.479134
6	-3.565585	-0.506381	-0.694415
6	-2.491429	1.548755	-0.248731
6	-3.623914	-1.866753	-0.674973
15	-2.196723	-2.674722	0.064338
15	-0.016281	1.329287	0.739319
6	-1.434189	2.288445	0.186612
1	-4.408473	0.073931	-1.100159
1	-4.503201	-2.392495	-1.049059
1	-3.392115	2.051239	-0.633573
1	-1.463574	3.378083	0.150538
6	1.405096	1.824132	-0.394961
6	-1.508794	-3.804890	-1.280854
15	0.885231	-1.909694	1.298523
6	2.602642	0.883616	-0.213644
6	1.836922	3.279037	-0.197175
6	0.867663	1.643487	-1.822690
1	2.331507	3.432145	0.772682
1	0.983895	3.970682	-0.269673
1	2.558793	3.554262	-0.983435
1	0.464332	0.629080	-1.973971
1	1.691984	1.792425	-2.538119

1	0.069764	2.362926	-2.050496
1	3.002078	0.888855	0.808734
1	3.410015	1.200315	-0.893978
1	2.334277	-0.153301	-0.462450
6	-0.571203	-4.873099	-0.711308
6	-0.704363	-2.864990	-2.193185
6	-2.617537	-4.462971	-2.108705
1	0.208082	-4.433930	-0.069667
1	-0.069392	-5.388118	-1.546498
1	-1.116625	-5.634487	-0.135217
1	-3.269034	-5.107011	-1.502846
1	-2.157910	-5.090990	-2.889225
1	-3.240412	-3.707505	-2.607504
1	0.180007	-2.469048	-1.670301
1	-1.319941	-2.016909	-2.533937
1	-0.360950	-3.418843	-3.081766
6	-2.864797	-3.628569	1.546472
6	-3.672877	-2.604967	2.358319
6	-1.719772	-4.139921	2.428908
6	-3.779564	-4.785158	1.137328
1	-1.120022	-3.306631	2.822777
1	-1.039662	-4.819300	1.899198
1	-2.147196	-4.687968	3.284447
1	-3.069393	-1.713304	2.593468
1	-3.984158	-3.066039	3.309140
1	-4.569682	-2.274273	1.817478
1	-4.579980	-4.450499	0.460125

1	-4.256669	-5.207589	2.036768
1	-3.221897	-5.596321	0.647817
6	0.267266	1.842508	2.532659
6	-0.781476	1.047213	3.326621
6	0.020546	3.338366	2.753844
6	1.665169	1.459804	3.026382
1	1.891606	0.399977	2.832963
1	1.714950	1.622000	4.115341
1	2.448085	2.079063	2.565202
1	-1.794383	1.210868	2.924651
1	-0.772832	1.374094	4.378920
1	-0.562734	-0.031512	3.298559
1	-1.012717	3.610945	2.497773
1	0.702633	3.968136	2.167048
1	0.178828	3.574829	3.818697

Table S3

[As-Rea][3]

E : -5266.12141534 Hartree

G : -5265.616362 Hartree

Atomic Number	Coordinates (Angstroms) X	Y	Z
7	-2.483047	0.182504	-0.223144
28	-0.915372	-0.791245	0.487990

6	-3.555665	-0.512535	-0.682806
6	-2.477945	1.540523	-0.247449
6	-3.618670	-1.871990	-0.661598
15	-2.192122	-2.686945	0.071329
15	-0.002262	1.329510	0.743334
6	-1.421396	2.282613	0.183354
1	-4.397525	0.070213	-1.086958
1	-4.500708	-2.394748	-1.033308
1	-3.379001	2.040500	-0.634444
1	-1.451982	3.371999	0.142111
6	1.415776	1.834767	-0.392272
6	-1.521485	-3.816575	-1.285605
33	0.994945	-1.978436	1.353063
6	2.629673	0.917250	-0.202809
6	1.822893	3.298301	-0.204811
6	0.884930	1.634986	-1.819938
1	2.313778	3.467089	0.764301
1	0.958606	3.974994	-0.283756
1	2.541003	3.579356	-0.992426
1	0.499338	0.612788	-1.965239
1	1.708505	1.792501	-2.534410
1	0.075826	2.339429	-2.054924
1	3.017775	0.922545	0.824043
1	3.438200	1.254443	-0.871803
1	2.385544	-0.122692	-0.463740
6	-0.603292	-4.909271	-0.731239
6	-0.703816	-2.881624	-2.191116

6	-2.642157	-4.447519	-2.119054
1	0.189685	-4.495124	-0.089954
1	-0.117034	-5.426409	-1.574263
1	-1.161283	-5.664757	-0.159468
1	-3.306801	-5.083150	-1.518630
1	-2.193216	-5.078957	-2.902990
1	-3.249566	-3.677054	-2.613657
1	0.190433	-2.506521	-1.669531
1	-1.305725	-2.019188	-2.520299
1	-0.374465	-3.431861	-3.087241
6	-2.871129	-3.646752	1.545873
6	-3.665032	-2.620954	2.368873
6	-1.735737	-4.185733	2.424099
6	-3.802609	-4.785445	1.123875
1	-1.135796	-3.365289	2.843721
1	-1.054681	-4.856388	1.884308
1	-2.172734	-4.751003	3.263421
1	-3.049067	-1.740722	2.614736
1	-3.983255	-3.088234	3.314363
1	-4.556839	-2.271603	1.831533
1	-4.597607	-4.432141	0.449895
1	-4.286426	-5.209814	2.018787
1	-3.256697	-5.599816	0.626566
6	0.272393	1.863527	2.533911
6	-0.764637	1.060252	3.335075
6	-0.000527	3.357322	2.740000
6	1.676228	1.514013	3.035205

1	1.924769	0.456152	2.859824
1	1.720939	1.693624	4.121623
1	2.448330	2.140668	2.565905
1	-1.779123	1.202690	2.928850
1	-0.764308	1.400203	4.383265
1	-0.529445	-0.015363	3.321836
1	-1.039631	3.608548	2.486307
1	0.667206	3.993222	2.143396
1	0.158622	3.607524	3.801549

Table S4

[Sb-Rea]^3

E : -3270.69589566 Hartree

G : -3270.193837 Hartree

Atomic Number	Coordinates (Angstroms) X	Y	Z
7	-2.469809	0.174478	-0.224119
28	-0.872533	-0.816197	0.494610
6	-3.540892	-0.523248	-0.668568
6	-2.454752	1.527401	-0.256125
6	-3.602425	-1.884762	-0.636924
15	-2.186463	-2.728332	0.085312
15	0.042921	1.340859	0.740345
6	-1.390828	2.266093	0.169120

1	-4.390845	0.049817	-1.072548
1	-4.489555	-2.406019	-0.999449
1	-3.351057	2.038347	-0.642872
1	-1.419946	3.355502	0.119319
6	1.453188	1.899941	-0.384245
6	-1.565655	-3.869221	-1.290810
51	1.271726	-2.146729	1.451809
6	2.688301	1.012042	-0.192772
6	1.823906	3.371924	-0.189199
6	0.938005	1.695051	-1.816783
1	2.305570	3.550221	0.782810
1	0.943158	4.026660	-0.269674
1	2.538254	3.674664	-0.972422
1	0.569474	0.667418	-1.968370
1	1.763659	1.869647	-2.525122
1	0.117816	2.385434	-2.054428
1	3.062752	1.010061	0.839743
1	3.499079	1.376300	-0.844684
1	2.471741	-0.028220	-0.477615
6	-0.680600	-4.998934	-0.757872
6	-0.724574	-2.950803	-2.191512
6	-2.707540	-4.455660	-2.127874
1	0.133515	-4.621519	-0.119951
1	-0.218749	-5.524422	-1.609505
1	-1.257028	-5.740599	-0.186414
1	-3.389403	-5.078258	-1.533247
1	-2.281443	-5.090293	-2.922133

1	-3.292563	-3.660163	-2.609197
1	0.184562	-2.607630	-1.673330
1	-1.300892	-2.065284	-2.504386
1	-0.417410	-3.499202	-3.096740
6	-2.897114	-3.696230	1.543265
6	-3.674287	-2.665854	2.376248
6	-1.774491	-4.264904	2.418875
6	-3.849444	-4.812956	1.109972
1	-1.172060	-3.456241	2.858768
1	-1.096610	-4.933396	1.871258
1	-2.219216	-4.840329	3.247214
1	-3.046689	-1.794786	2.625642
1	-3.998045	-3.134806	3.319221
1	-4.561098	-2.299977	1.841892
1	-4.636135	-4.436807	0.438818
1	-4.343412	-5.236663	1.999845
1	-3.320143	-5.633323	0.604678
6	0.289543	1.910613	2.528077
6	-0.729332	1.085613	3.330313
6	-0.030364	3.396758	2.720841
6	1.698759	1.611502	3.045730
1	1.982182	0.559084	2.889736
1	1.731817	1.807345	4.129830
1	2.456972	2.251480	2.571900
1	-1.743505	1.192819	2.912962
1	-0.748535	1.434544	4.375501
1	-0.465374	0.016344	3.330355

1	-1.074144	3.613244	2.455372
1	0.622634	4.048401	2.125112
1	0.111002	3.660356	3.781835

Table S5

[Bi-Rea]^3

E : -3245.04408674 Hartree

G : -3244.543396 Hartree

Atomic Number	Coordinates (Angstroms) X	Y	Z
7	-2.480957	0.180800	-0.223975
28	-0.874976	-0.816023	0.501646
6	-3.550432	-0.520652	-0.660471
6	-2.456874	1.531502	-0.261415
6	-3.609173	-1.883897	-0.622055
15	-2.195116	-2.735667	0.095973
15	0.050195	1.347546	0.737005
6	-1.386528	2.266182	0.160395
1	-4.404932	0.045935	-1.065479
1	-4.498137	-2.405552	-0.980208
1	-3.349161	2.049929	-0.649056
1	-1.412254	3.355604	0.104580
6	1.462383	1.910374	-0.384312
6	-1.576959	-3.868810	-1.288997

83	1.382083	-2.217035	1.516546
6	2.688763	1.009757	-0.193907
6	1.845474	3.378378	-0.185058
6	0.948025	1.714706	-1.818308
1	2.325762	3.551370	0.788580
1	0.969670	4.039711	-0.265792
1	2.563855	3.678159	-0.965858
1	0.566709	0.692173	-1.972833
1	1.776529	1.881583	-2.525330
1	0.135882	2.414808	-2.054953
1	3.061896	1.002038	0.839399
1	3.505138	1.364327	-0.844247
1	2.459463	-0.027755	-0.481156
6	-0.686135	-4.996904	-0.762646
6	-0.737730	-2.942576	-2.183401
6	-2.715747	-4.454521	-2.130148
1	0.127104	-4.616884	-0.124117
1	-0.221594	-5.517148	-1.616105
1	-1.256751	-5.743605	-0.191980
1	-3.391857	-5.087961	-1.540460
1	-2.288212	-5.077514	-2.932949
1	-3.307448	-3.657269	-2.600374
1	0.170050	-2.600340	-1.661386
1	-1.315420	-2.055796	-2.489840
1	-0.427407	-3.483441	-3.092183
6	-2.909460	-3.719506	1.542004
6	-3.700527	-2.701666	2.377098

6	-1.781640	-4.277380	2.418261
6	-3.848243	-4.845115	1.102981
1	-1.189737	-3.460724	2.859044
1	-1.095561	-4.936918	1.869361
1	-2.218042	-4.859263	3.246568
1	-3.085226	-1.822303	2.628003
1	-4.019291	-3.176568	3.318912
1	-4.591169	-2.346131	1.842212
1	-4.637479	-4.474224	0.431859
1	-4.339733	-5.278473	1.989690
1	-3.309700	-5.657433	0.594464
6	0.293588	1.929216	2.522176
6	-0.726791	1.106979	3.325272
6	-0.024415	3.415948	2.710984
6	1.700659	1.627734	3.044013
1	1.979220	0.572655	2.893463
1	1.733935	1.827848	4.127379
1	2.463457	2.260522	2.567988
1	-1.740126	1.212572	2.905733
1	-0.747538	1.457049	4.370131
1	-0.463218	0.037258	3.327044
1	-1.065800	3.633352	2.436851
1	0.633956	4.065033	2.118413
1	0.109278	3.682202	3.772411

Table S6

[N-Rea][1]

E : -3085.05018650 Hartree

G : -3084.540298 Hartree

Atomic Number	Coordinates (Angstroms) X	Y	Z
7	-2.596303	0.252045	-0.272526
28	-1.027457	-0.722229	0.438618
6	-3.665871	-0.461764	-0.701110
6	-2.557709	1.606300	-0.312224
6	-3.694472	-1.825881	-0.653459
15	-2.222795	-2.595786	0.035801
15	-0.102741	1.318376	0.721884
6	-1.473362	2.314551	0.119712
1	-4.527724	0.095091	-1.099611
1	-4.566941	-2.381970	-0.998074
1	-3.440298	2.133215	-0.706022
1	-1.459617	3.404034	0.074617
6	1.391773	1.669619	-0.361893
6	-1.481592	-3.664173	-1.317519
7	0.304116	-1.548784	1.041123
6	2.494927	0.638977	-0.087826
6	1.923758	3.094048	-0.192982
6	0.891223	1.471530	-1.800673
1	2.383540	3.241526	0.794742
1	1.131537	3.846385	-0.328890

1	2.700913	3.284339	-0.950826
1	0.463618	0.465622	-1.935440
1	1.741505	1.577700	-2.492477
1	0.123326	2.209436	-2.070483
1	2.993513	0.806640	0.874832
1	3.260533	0.719547	-0.876253
1	2.089689	-0.385952	-0.092214
6	-0.385508	-4.563329	-0.741564
6	-0.836808	-2.667293	-2.293206
6	-2.541665	-4.482472	-2.059238
1	0.310524	-3.979973	-0.116306
1	0.189401	-5.005346	-1.571069
1	-0.801703	-5.390328	-0.148800
1	-3.065266	-5.188854	-1.401436
1	-2.053737	-5.065780	-2.856804
1	-3.287180	-3.826391	-2.531312
1	0.010244	-2.157320	-1.811136
1	-1.560728	-1.909933	-2.633575
1	-0.466717	-3.212302	-3.176362
6	-2.732419	-3.558037	1.567465
6	-3.529304	-2.555396	2.415788
6	-1.489066	-3.982130	2.360361
6	-3.609186	-4.769024	1.242512
1	-0.796094	-3.135654	2.494113
1	-0.942095	-4.799266	1.873949
1	-1.805622	-4.340007	3.353321
1	-2.933806	-1.652743	2.624278

1	-3.786478	-3.026022	3.377650
1	-4.457234	-2.245660	1.915803
1	-4.477453	-4.493119	0.624333
1	-3.990074	-5.204161	2.180648
1	-3.041663	-5.553829	0.722062
6	0.147974	1.769079	2.526596
6	-1.023926	1.100495	3.262260
6	0.094812	3.280865	2.761870
6	1.456916	1.169731	3.045351
1	1.534941	0.104545	2.771358
1	1.474151	1.241632	4.144680
1	2.335628	1.709224	2.664098
1	-1.993316	1.398889	2.832522
1	-1.007715	1.404208	4.321240
1	-0.932834	0.005699	3.206777
1	-0.874076	3.694887	2.447239
1	0.892555	3.818595	2.232301
1	0.213026	3.482272	3.838845

Table S7

[P-Rea][1]

E : -3371.67530221 Hartree

G : -3371.167080 Hartree

Atomic	Coordinates (Angstroms)		
Number	X	Y	Z

7	-2.544478	0.219357	-0.245887
28	-0.988982	-0.747531	0.463951
6	-3.662232	-0.479864	-0.561871
6	-2.464319	1.563620	-0.403366
6	-3.726247	-1.835563	-0.441107
15	-2.211750	-2.634475	0.117363
15	-0.024838	1.300551	0.686340
6	-1.353795	2.268918	-0.048701
1	-4.532389	0.084662	-0.929753
1	-4.634810	-2.380982	-0.698770
1	-3.336657	2.082135	-0.829278
1	-1.306537	3.349938	-0.184858
6	1.530087	1.668624	-0.304212
6	-1.627788	-3.650942	-1.362354
15	0.687502	-1.791758	1.231083
6	2.671075	0.732331	0.107574
6	1.970643	3.129396	-0.177955
6	1.160025	1.364742	-1.763448
1	2.352869	3.355399	0.827749
1	1.150324	3.826116	-0.407788
1	2.785914	3.324926	-0.892996
1	0.805033	0.327934	-1.871280
1	2.054933	1.488021	-2.393636
1	0.374129	2.038358	-2.131946
1	3.004551	0.892224	1.140336
1	3.535467	0.908856	-0.552849

1	2.380802	-0.326391	-0.014827
6	-0.574886	-4.685070	-0.960286
6	-0.979222	-2.626300	-2.306745
6	-2.794759	-4.326902	-2.089554
1	0.234099	-4.225094	-0.368915
1	-0.121290	-5.103607	-1.873072
1	-1.011093	-5.521479	-0.395412
1	-3.339096	-5.035912	-1.451509
1	-2.399744	-4.888282	-2.951558
1	-3.506943	-3.581972	-2.470868
1	-0.053750	-2.228596	-1.864885
1	-1.659734	-1.786296	-2.519737
1	-0.732600	-3.119526	-3.260778
6	-2.669097	-3.693664	1.602203
6	-3.364898	-2.737068	2.581582
6	-1.412544	-4.247292	2.282146
6	-3.620016	-4.835285	1.233168
1	-0.733977	-3.431108	2.588149
1	-0.858603	-4.952083	1.649941
1	-1.707557	-4.782740	3.199073
1	-2.709865	-1.888758	2.834470
1	-3.595797	-3.280841	3.511211
1	-4.300177	-2.338467	2.165029
1	-4.504211	-4.472183	0.687693
1	-3.973328	-5.324856	2.154950
1	-3.120670	-5.601488	0.623162
6	0.078438	1.900929	2.473122

6	-1.094268	1.207969	3.185117
6	-0.103426	3.418542	2.578633
6	1.384872	1.469012	3.141299
1	1.567170	0.390015	3.005762
1	1.310096	1.658889	4.224092
1	2.248730	2.036167	2.765642
1	-2.046380	1.383209	2.658973
1	-1.186450	1.611601	4.206318
1	-0.919302	0.123860	3.247942
1	-1.084905	3.726375	2.192049
1	0.674434	3.977584	2.041518
1	-0.052196	3.710310	3.640000

Table S8

[As-Rea][1]

E : -5266.08790675 Hartree

G : -5265.580468 Hartree

Atomic Number	Coordinates (Angstroms) X	Y	Z
7	-2.525065	0.207596	-0.237825
28	-0.972427	-0.758167	0.470430
6	-3.663138	-0.480311	-0.510355
6	-2.433492	1.546415	-0.442437
6	-3.748597	-1.831652	-0.364588

15	-2.221220	-2.641254	0.138642
15	-0.008631	1.303096	0.667976
6	-1.317384	2.256724	-0.119427
1	-4.532147	0.093996	-0.865155
1	-4.672804	-2.364307	-0.591239
1	-3.305327	2.054896	-0.880931
1	-1.266053	3.332403	-0.291434
6	1.576861	1.654432	-0.276326
6	-1.690272	-3.635036	-1.379280
33	0.818286	-1.872281	1.288604
6	2.689743	0.705084	0.181287
6	2.031557	3.110153	-0.142035
6	1.255109	1.348952	-1.746347
1	2.376263	3.340008	0.876142
1	1.228730	3.814157	-0.408412
1	2.875933	3.291312	-0.826467
1	0.885137	0.318275	-1.863223
1	2.173921	1.452928	-2.344943
1	0.495097	2.034512	-2.145743
1	2.956579	0.831167	1.237916
1	3.596027	0.893341	-0.416717
1	2.409774	-0.349482	-0.000140
6	-0.640836	-4.694526	-1.037617
6	-1.055714	-2.596117	-2.317609
6	-2.886265	-4.279406	-2.088117
1	0.202227	-4.260033	-0.476068
1	-0.236519	-5.107190	-1.975948

1	-1.067796	-5.532159	-0.467283
1	-3.419587	-4.998166	-1.451430
1	-2.522986	-4.824096	-2.974481
1	-3.600993	-3.518229	-2.430225
1	-0.114227	-2.217278	-1.892483
1	-1.732417	-1.745004	-2.495346
1	-0.837601	-3.070090	-3.288224
6	-2.630525	-3.731280	1.613105
6	-3.319884	-2.807821	2.627887
6	-1.343833	-4.270440	2.247563
6	-3.568298	-4.885179	1.248553
1	-0.701456	-3.442802	2.602349
1	-0.765733	-4.911977	1.570979
1	-1.600200	-4.868209	3.137125
1	-2.680644	-1.945534	2.874383
1	-3.508846	-3.370369	3.555895
1	-4.277251	-2.427959	2.245400
1	-4.474524	-4.527661	0.736590
1	-3.885656	-5.400026	2.169673
1	-3.073732	-5.628746	0.607609
6	0.040827	1.945777	2.445062
6	-1.142936	1.252682	3.138503
6	-0.164472	3.462757	2.510120
6	1.331849	1.552186	3.165349
1	1.527135	0.470832	3.079609
1	1.224209	1.785614	4.236870
1	2.201800	2.112178	2.792730

1	-2.082525	1.405858	2.583830
1	-1.268138	1.674040	4.149004
1	-0.957651	0.171902	3.229101
1	-1.138074	3.748833	2.088805
1	0.621482	4.019854	1.982642
1	-0.147086	3.779018	3.565563

Table S9

[Sb-Rea][1]

E : -3270.65687596 Hartree

G : -3270.149779 Hartree

Atomic Number	Coordinates (Angstroms) X	Y	Z
7	-2.499424	0.192236	-0.227491
28	-0.947973	-0.772694	0.480127
6	-3.652609	-0.485079	-0.467214
6	-2.401318	1.525696	-0.468906
6	-3.755238	-1.832396	-0.304297
15	-2.220035	-2.653765	0.152991
15	0.010084	1.304034	0.661194
6	-1.283125	2.241417	-0.169401
1	-4.520062	0.098133	-0.810851
1	-4.691141	-2.353307	-0.509009
1	-3.273233	2.024193	-0.918352

1	-1.231530	3.312373	-0.368385
6	1.614559	1.652563	-0.251674
6	-1.746102	-3.636358	-1.394728
51	1.029852	-2.003345	1.382375
6	2.717902	0.708480	0.240856
6	2.064992	3.109721	-0.117399
6	1.331419	1.337714	-1.727468
1	2.380701	3.350567	0.907438
1	1.267974	3.808889	-0.412032
1	2.926873	3.286641	-0.780851
1	0.955865	0.309245	-1.848279
1	2.266540	1.430129	-2.302397
1	0.587857	2.026172	-2.151861
1	2.915573	0.795463	1.316911
1	3.657369	0.931508	-0.289914
1	2.474267	-0.341850	-0.011523
6	-0.718082	-4.732153	-1.105407
6	-1.109339	-2.594365	-2.328037
6	-2.974259	-4.236709	-2.087417
1	0.161492	-4.332940	-0.575539
1	-0.366143	-5.146795	-2.063782
1	-1.147735	-5.563024	-0.527075
1	-3.510770	-4.954277	-1.451931
1	-2.644512	-4.773522	-2.991511
1	-3.677433	-3.452237	-2.398822
1	-0.151098	-2.241320	-1.917684
1	-1.772096	-1.727071	-2.477537

1	-0.919511	-3.054908	-3.310982
6	-2.605013	-3.766353	1.617270
6	-3.278399	-2.864078	2.661162
6	-1.305560	-4.314982	2.218352
6	-3.547186	-4.916571	1.251774
1	-0.695017	-3.494020	2.642029
1	-0.706304	-4.892662	1.503000
1	-1.542783	-4.977246	3.066390
1	-2.643206	-1.997825	2.905258
1	-3.440908	-3.440439	3.585791
1	-4.247790	-2.488852	2.305538
1	-4.463912	-4.550443	0.765462
1	-3.845337	-5.448229	2.169796
1	-3.066175	-5.647451	0.586635
6	0.017789	1.993630	2.424566
6	-1.164286	1.292961	3.113165
6	-0.222808	3.507007	2.446199
6	1.302102	1.655438	3.184449
1	1.517742	0.575440	3.156163
1	1.170585	1.938141	4.241360
1	2.170059	2.212080	2.802018
1	-2.096975	1.416781	2.539902
1	-1.314911	1.732036	4.112597
1	-0.961709	0.217661	3.231561
1	-1.195042	3.760859	2.002238
1	0.559551	4.068056	1.917311
1	-0.229470	3.850195	3.493317

Table S10

[Bi-Rea][1]

E : -3244.99560157 Hartree

G : -3244.488653 Hartree

Atomic Number	Coordinates (Angstroms) X	Y	Z
7	-2.502288	0.194050	-0.228412
28	-0.951896	-0.769911	0.478034
6	-3.658140	-0.482348	-0.460545
6	-2.401630	1.526412	-0.477495
6	-3.763598	-1.829519	-0.294961
15	-2.223723	-2.650211	0.148583
15	0.003623	1.307230	0.660531
6	-1.282734	2.243562	-0.182219
1	-4.526063	0.102274	-0.800720
1	-4.702169	-2.347248	-0.495653
1	-3.273104	2.023085	-0.929912
1	-1.232280	3.313541	-0.386818
6	1.618257	1.639501	-0.240190
6	-1.762724	-3.632454	-1.403694
83	1.095280	-2.043687	1.409950
6	2.702904	0.676467	0.260424
6	2.089332	3.089391	-0.099367

6	1.344043	1.332897	-1.719262
1	2.397474	3.325591	0.928763
1	1.304846	3.799537	-0.401340
1	2.960155	3.255128	-0.754067
1	0.950498	0.311878	-1.846495
1	2.285867	1.409674	-2.285606
1	0.616974	2.035654	-2.148724
1	2.875392	0.741011	1.342595
1	3.657226	0.899851	-0.242837
1	2.456243	-0.365580	-0.025022
6	-0.729221	-4.726552	-1.126612
6	-1.136019	-2.588008	-2.341029
6	-2.996003	-4.234514	-2.085408
1	0.157612	-4.324057	-0.611690
1	-0.392032	-5.143941	-2.089135
1	-1.148394	-5.556452	-0.539221
1	-3.523318	-4.955539	-1.446043
1	-2.674425	-4.767914	-2.994492
1	-3.705049	-3.451492	-2.387032
1	-0.176478	-2.231507	-1.936240
1	-1.802886	-1.723231	-2.486247
1	-0.949809	-3.047318	-3.325256
6	-2.586201	-3.766028	1.615963
6	-3.264955	-2.873306	2.664347
6	-1.272065	-4.293512	2.206843
6	-3.514104	-4.930813	1.260645
1	-0.687145	-3.463453	2.650740

1	-0.658527	-4.841783	1.480190
1	-1.490068	-4.977807	3.042380
1	-2.643404	-1.994759	2.899763
1	-3.408590	-3.449500	3.592268
1	-4.244091	-2.516281	2.316826
1	-4.440467	-4.577544	0.783150
1	-3.796017	-5.466421	2.181564
1	-3.029842	-5.654862	0.590549
6	0.004870	2.007237	2.420102
6	-1.184114	1.314175	3.104420
6	-0.229085	3.521628	2.432437
6	1.282103	1.667102	3.191305
1	1.487606	0.584944	3.176078
1	1.146911	1.963486	4.244031
1	2.157486	2.211441	2.808047
1	-2.113347	1.441229	2.526426
1	-1.337537	1.755244	4.102558
1	-0.986912	0.238008	3.225867
1	-1.195827	3.778033	1.978030
1	0.561029	4.076180	1.908085
1	-0.243863	3.870306	3.477692

Table S11

CO_2

E : -188.370468444 Hartree

G : -188.379676 Hartree

Atomic	Coordinates (Angstroms)		
Number	X	Y	Z
6	0.000000	0.000000	-0.000000
8	0.000000	0.000000	-1.156133
8	-0.000000	-0.000000	1.156133

Table S12

[N-TS]3

E : -3273.47339061 Hartree

G : -3272.956102 Hartree

Atomic	Coordinates (Angstroms)		
Number	X	Y	Z
7	-2.655321	0.193914	0.564030
28	-0.955938	-0.758006	0.563788
6	-3.821104	-0.507898	0.488529
6	-2.665065	1.554001	0.650486
6	-3.863844	-1.866901	0.427516
15	-2.255876	-2.665236	0.499480
15	-0.012904	1.344481	0.590567
6	-1.528217	2.301322	0.703997
1	-4.754037	0.074574	0.474858
1	-4.816317	-2.394566	0.369463

1	-3.649157	2.044522	0.679164
1	-1.575369	3.389044	0.763919
6	0.891796	1.875931	-0.965540
6	-2.057454	-3.643506	-1.091802
7	0.679165	-1.639240	0.584858
6	1.928082	0.806087	-1.336815
6	1.548808	3.251449	-0.833954
6	-0.171700	1.922140	-2.072253
1	2.387277	3.240336	-0.123700
1	0.826694	4.018046	-0.513437
1	1.948872	3.556973	-1.814265
1	-0.739491	0.979123	-2.124674
1	0.328784	2.071148	-3.041927
1	-0.887060	2.740786	-1.916124
1	2.664457	0.619650	-0.543885
1	2.473890	1.131937	-2.236746
1	1.437378	-0.150524	-1.571876
6	-0.924223	-4.666421	-0.982636
6	-1.665956	-2.589138	-2.140334
6	-3.364493	-4.322491	-1.509776
1	0.010002	-4.198754	-0.638492
1	-0.729167	-5.083896	-1.983350
1	-1.185611	-5.503722	-0.319869
1	-3.718784	-5.043336	-0.759572
1	-3.199785	-4.871335	-2.450947
1	-4.158495	-3.582603	-1.684535
1	-0.651916	-2.206953	-1.942330

1	-2.379600	-1.749315	-2.155702
1	-1.662015	-3.051939	-3.140017
6	-2.222695	-3.727841	2.047471
6	-2.692191	-2.808239	3.184576
6	-0.781980	-4.166354	2.343762
6	-3.152506	-4.939284	1.954444
1	-0.103463	-3.301314	2.395550
1	-0.386996	-4.857572	1.589327
1	-0.758144	-4.682191	3.317079
1	-2.082465	-1.891935	3.238935
1	-2.589220	-3.341456	4.142695
1	-3.741988	-2.510240	3.059775
1	-4.182047	-4.640814	1.703910
1	-3.180358	-5.454685	2.928255
1	-2.806568	-5.665349	1.205201
6	0.961219	1.647053	2.167236
6	0.222356	0.823880	3.234996
6	0.954452	3.122267	2.577724
6	2.394326	1.121291	2.041970
1	2.427363	0.076798	1.694358
1	2.873044	1.160891	3.033680
1	3.001198	1.735756	1.361452
1	-0.847853	1.082561	3.276713
1	0.663193	1.026408	4.223987
1	0.321273	-0.254248	3.032783
1	-0.071251	3.487382	2.731898
1	1.446711	3.764752	1.835494

1	1.498935	3.233496	3.529332
6	2.356441	-2.280752	-0.254077
8	1.933881	-2.784863	-1.236948
8	3.217418	-1.928590	0.468336

Table S13

[P-TS][3]

E : -3560.05405079 Hartree

G : -3559.541049 Hartree

Atomic Number	Coordinates (Angstroms) X	Y	Z
7	-2.513515	0.139637	0.581386
28	-0.771888	-0.775614	0.784069
6	-3.667778	-0.578541	0.693615
6	-2.565423	1.494317	0.474216
6	-3.699352	-1.932807	0.770183
15	-2.116783	-2.773844	0.590732
15	0.099823	1.427454	0.647699
6	-1.461966	2.290012	0.464218
1	-4.607071	-0.006822	0.717683
1	-4.653341	-2.456082	0.842539
1	-3.564805	1.947296	0.395080
1	-1.557231	3.371022	0.360164
6	1.109579	1.767822	-0.900330

6	-2.224046	-3.520005	-1.147134
15	1.228332	-1.771192	1.521512
6	2.346255	0.861424	-0.945529
6	1.510689	3.238730	-1.027113
6	0.190185	1.380488	-2.068407
1	2.254255	3.527491	-0.270410
1	0.641773	3.908996	-0.941542
1	1.966024	3.404901	-2.016767
1	-0.158708	0.339576	-1.973034
1	0.756462	1.457283	-3.009725
1	-0.688765	2.036617	-2.131088
1	3.033568	1.032742	-0.107098
1	2.899463	1.066475	-1.876245
1	2.056504	-0.198620	-0.956685
6	-1.229296	-4.663437	-1.360568
6	-1.875882	-2.354048	-2.086610
6	-3.642430	-4.005013	-1.468038
1	-0.205019	-4.383036	-1.080434
1	-1.218058	-4.919875	-2.432107
1	-1.525738	-5.568427	-0.810864
1	-3.992467	-4.780991	-0.773559
1	-3.644033	-4.437867	-2.481396
1	-4.361374	-3.174819	-1.454357
1	-0.815107	-2.083756	-1.990587
1	-2.501190	-1.470421	-1.878906
1	-2.060804	-2.661696	-3.128484
6	-2.062883	-4.072773	1.950040

6	-2.200215	-3.283253	3.260228
6	-0.722757	-4.818775	1.967146
6	-3.209705	-5.080643	1.832187
1	0.128687	-4.136609	2.104107
1	-0.545497	-5.401324	1.055504
1	-0.721528	-5.519536	2.817475
1	-1.387576	-2.546270	3.365066
1	-2.135591	-3.979760	4.110990
1	-3.160165	-2.750483	3.315983
1	-4.190358	-4.586059	1.769583
1	-3.216743	-5.725866	2.725522
1	-3.087402	-5.732930	0.955793
6	0.870826	2.137686	2.215648
6	0.118252	1.451421	3.366442
6	0.653040	3.651262	2.320998
6	2.363974	1.817263	2.322749
1	2.568583	0.743968	2.189177
1	2.717433	2.103268	3.326337
1	2.958041	2.381447	1.589880
1	-0.971326	1.574226	3.261175
1	0.421926	1.903432	4.324114
1	0.346934	0.375634	3.410777
1	-0.417194	3.896918	2.356854
1	1.110063	4.200703	1.487108
1	1.113271	4.013900	3.254260
6	1.857945	-2.923077	-0.054542
8	1.145583	-2.384841	-0.904959

8	2.631931	-3.817808	0.074289

Table S14

[As-TS]^3

E : -5454.46615616 Hartree

G : -5453.954200 Hartree

Atomic Number	Coordinates (Angstroms) X	Y	Z
7	-2.425753	0.125768	0.232386
28	-0.714000	-0.867320	0.354649
6	-3.606719	-0.550013	0.168134
6	-2.433484	1.487410	0.270211
6	-3.713279	-1.903604	0.219114
15	-2.177235	-2.822335	0.372041
15	0.235870	1.367184	0.573587
6	-1.320204	2.251126	0.425439
1	-4.517436	0.061012	0.082796
1	-4.694496	-2.376434	0.158637
1	-3.415461	1.974875	0.179960
1	-1.401376	3.338823	0.441377
6	1.359440	2.077611	-0.759699
6	-2.189580	-4.067124	-1.041021
33	1.523305	-2.204405	0.546094
6	2.632617	1.235497	-0.902850

6	1.721993	3.541121	-0.496702
6	0.563273	1.973193	-2.069714
1	2.390197	3.651646	0.369265
1	0.828383	4.162412	-0.333492
1	2.251477	3.942636	-1.375855
1	0.251252	0.936013	-2.263580
1	1.210216	2.295504	-2.901078
1	-0.328026	2.615274	-2.053875
1	3.245690	1.223246	0.007313
1	3.247469	1.656153	-1.714548
1	2.392924	0.199668	-1.174932
6	-0.835146	-4.774358	-1.172753
6	-2.430742	-3.243320	-2.315261
6	-3.304273	-5.104766	-0.883341
1	-0.028683	-4.069089	-1.410397
1	-0.893058	-5.492569	-2.006312
1	-0.551327	-5.333662	-0.272297
1	-3.108111	-5.796486	-0.051532
1	-3.366905	-5.705776	-1.804895
1	-4.286078	-4.633471	-0.725548
1	-1.674816	-2.451311	-2.426004
1	-3.428301	-2.783002	-2.316507
1	-2.354343	-3.912413	-3.187038
6	-2.278986	-3.650103	2.068733
6	-1.907115	-2.533043	3.055327
6	-1.265726	-4.789431	2.204459
6	-3.688425	-4.149026	2.400903

1	-0.254167	-4.474636	1.902341
1	-1.552021	-5.668542	1.609900
1	-1.218475	-5.104058	3.259532
1	-0.850008	-2.246891	2.941300
1	-2.054739	-2.884903	4.089043
1	-2.535054	-1.639392	2.906537
1	-4.411384	-3.321395	2.394694
1	-3.685174	-4.587655	3.412085
1	-4.037512	-4.921351	1.703133
6	0.848681	1.750768	2.319105
6	0.023312	0.821717	3.221754
6	0.584149	3.200955	2.734583
6	2.332305	1.410842	2.481398
1	2.559633	0.395319	2.119747
1	2.596517	1.454368	3.550344
1	2.978714	2.124779	1.951687
1	-1.057614	0.947327	3.046719
1	0.226730	1.053721	4.279560
1	0.290574	-0.232008	3.047364
1	-0.490483	3.429530	2.705748
1	1.110823	3.924323	2.098601
1	0.933291	3.347965	3.769647
6	1.388277	-2.066040	-1.657703
8	0.353534	-1.382981	-1.653961
8	2.153456	-2.568385	-2.408340

Table S15

[Sb-TS]3

E : -3459.04210366 Hartree

G : -3458.530293 Hartree

Atomic Number	Coordinates (Angstroms) X	Y	Z
7	-2.402759	0.108683	0.246957
28	-0.666805	-0.905963	0.326573
6	-3.585616	-0.559384	0.177992
6	-2.403205	1.468993	0.246505
6	-3.699011	-1.913072	0.224267
15	-2.170595	-2.843252	0.379264
15	0.271582	1.349904	0.547815
6	-1.283993	2.230021	0.370884
1	-4.494710	0.054650	0.091120
1	-4.682557	-2.381426	0.166137
1	-3.382197	1.962200	0.149973
1	-1.359665	3.318316	0.365780
6	1.418460	2.092816	-0.750986
6	-2.208939	-4.106304	-1.019755
51	1.682521	-2.284269	0.661902
6	2.694046	1.254182	-0.893215
6	1.784226	3.547698	-0.444541
6	0.645107	2.039317	-2.077272
1	2.442377	3.632767	0.431516

1	0.891416	4.168311	-0.275736
1	2.325329	3.969426	-1.307082
1	0.282610	1.023648	-2.293620
1	1.319043	2.344899	-2.893459
1	-0.218952	2.717477	-2.066809
1	3.262290	1.175364	0.043033
1	3.348456	1.723348	-1.645363
1	2.473455	0.235230	-1.239188
6	-0.842960	-4.785248	-1.173143
6	-2.507067	-3.310495	-2.299461
6	-3.298059	-5.164501	-0.824844
1	-0.059725	-4.061064	-1.434902
1	-0.898847	-5.522053	-1.990500
1	-0.522232	-5.314404	-0.266073
1	-3.072384	-5.841156	0.011172
1	-3.367625	-5.778186	-1.737637
1	-4.285316	-4.709803	-0.653325
1	-1.797736	-2.480744	-2.434010
1	-3.524152	-2.895852	-2.287749
1	-2.414852	-3.984879	-3.165752
6	-2.287189	-3.661604	2.081624
6	-1.920121	-2.541874	3.067119
6	-1.285888	-4.809523	2.234309
6	-3.703436	-4.148205	2.405476
1	-0.265505	-4.501385	1.957443
1	-1.565535	-5.685540	1.631763
1	-1.264078	-5.127043	3.289332

1	-0.862077	-2.258370	2.961620
1	-2.080503	-2.891367	4.099752
1	-2.545134	-1.647772	2.908938
1	-4.421209	-3.316423	2.384151
1	-3.709771	-4.573161	3.422403
1	-4.052206	-4.928348	1.716720
6	0.837303	1.748200	2.309707
6	-0.001787	0.816522	3.197281
6	0.540660	3.196716	2.711233
6	2.321097	1.434729	2.518000
1	2.573374	0.414457	2.188984
1	2.554872	1.506696	3.592488
1	2.971735	2.146469	1.990144
1	-1.078789	0.928358	2.990696
1	0.169261	1.063390	4.257483
1	0.282064	-0.235042	3.040462
1	-0.535238	3.411425	2.648770
1	1.078915	3.927256	2.093931
1	0.854452	3.347368	3.756980
6	1.234861	-1.898368	-1.972704
8	0.203610	-1.328987	-1.707838
8	2.013691	-2.297279	-2.746724

Table S16

[Bi-TS]^3

E : -3433.38277576 Hartree

G : -3432.871989 Hartree

Atomic Number	Coordinates (Angstroms) X	Y	Z
7	-2.405273	0.109015	0.236134
28	-0.667484	-0.905079	0.318223
6	-3.586696	-0.561200	0.163653
6	-2.404785	1.469335	0.231589
6	-3.697543	-1.915140	0.211756
15	-2.169201	-2.844904	0.375667
15	0.270185	1.350921	0.549098
6	-1.285259	2.229592	0.358733
1	-4.496702	0.050866	0.072182
1	-4.680172	-2.385109	0.150957
1	-3.382847	1.963257	0.129160
1	-1.360304	3.317933	0.350566
6	1.426530	2.091639	-0.742347
6	-2.198150	-4.108292	-1.023198
83	1.745136	-2.317877	0.711010
6	2.702685	1.251957	-0.872614
6	1.790474	3.546988	-0.435912
6	0.663404	2.036138	-2.074409
1	2.441796	3.633458	0.445068
1	0.896650	4.168308	-0.275282
1	2.338481	3.966781	-1.295018
1	0.300986	1.020535	-2.291297

1	1.343836	2.338965	-2.886205
1	-0.200003	2.715213	-2.072095
1	3.262597	1.173824	0.068773
1	3.364070	1.719357	-1.619745
1	2.484200	0.232384	-1.218566
6	-0.829591	-4.783909	-1.167500
6	-2.490465	-3.313508	-2.304848
6	-3.286297	-5.168627	-0.834777
1	-0.046074	-4.056841	-1.420743
1	-0.876946	-5.519093	-1.986847
1	-0.514713	-5.314022	-0.258867
1	-3.065632	-5.843298	0.004126
1	-3.347448	-5.784075	-1.746980
1	-4.275847	-4.715780	-0.671740
1	-1.782377	-2.482073	-2.435575
1	-3.508459	-2.900963	-2.299146
1	-2.391688	-3.987768	-3.170502
6	-2.295690	-3.662543	2.077631
6	-1.935227	-2.542776	3.065490
6	-1.295317	-4.810444	2.236544
6	-3.714050	-4.149285	2.392247
1	-0.273273	-4.502175	1.965351
1	-1.571569	-5.686891	1.633060
1	-1.279294	-5.127093	3.291908
1	-0.876050	-2.260273	2.968158
1	-2.104023	-2.892017	4.096861
1	-2.558437	-1.648286	2.902361

1	-4.431343	-3.317152	2.368869
1	-3.726124	-4.576407	3.408186
1	-4.059389	-4.927807	1.699918
6	0.821294	1.754040	2.314651
6	-0.023948	0.823857	3.197934
6	0.519807	3.203395	2.709607
6	2.303744	1.442958	2.535920
1	2.559646	0.421801	2.211975
1	2.528347	1.517853	3.612141
1	2.958083	2.154016	2.011709
1	-1.099345	0.933353	2.981876
1	0.137726	1.074538	4.258705
1	0.263258	-0.227773	3.046973
1	-0.555858	3.416572	2.638761
1	1.061771	3.932922	2.094356
1	0.825703	3.357269	3.757199
6	1.248038	-1.895628	-1.988075
8	0.221644	-1.336943	-1.689755
8	2.017189	-2.281499	-2.777033

Table S17

[N-Int]^3

E : -3273.48492323 Hartree

G : -3272.966289 Hartree

Atomic Coordinates (Angstroms)

Number	X	Y	Z
7	-2.475098	0.216062	-0.247213
28	-0.993785	-0.762343	0.540575
6	-3.580268	-0.457483	-0.681620
6	-2.438362	1.579070	-0.288874
6	-3.690526	-1.810625	-0.637431
15	-2.259538	-2.670388	0.028814
15	-0.002641	1.345157	0.773146
6	-1.382356	2.311913	0.155047
1	-4.403362	0.150157	-1.084091
1	-4.589629	-2.308364	-1.001887
1	-3.322233	2.084431	-0.703944
1	-1.391219	3.400247	0.089493
6	1.488966	1.711088	-0.303818
6	-1.559689	-3.682985	-1.394420
7	0.394954	-1.595630	1.508693
6	2.556009	0.629005	-0.089427
6	2.064384	3.106429	-0.050725
6	0.988400	1.618477	-1.752705
1	2.534214	3.182926	0.939898
1	1.295648	3.889589	-0.138395
1	2.842425	3.314908	-0.802527
1	0.472988	0.662773	-1.940512
1	1.854357	1.672409	-2.430747
1	0.296512	2.435011	-1.999975
1	2.873040	0.527317	0.956815

1	3.446086	0.885336	-0.685821
1	2.201595	-0.355137	-0.428111
6	-0.599752	-4.769975	-0.904065
6	-0.768768	-2.660339	-2.227429
6	-2.672860	-4.293403	-2.250868
1	0.195297	-4.348382	-0.272289
1	-0.114066	-5.224967	-1.782133
1	-1.124866	-5.572916	-0.366773
1	-3.309851	-4.984182	-1.680746
1	-2.214138	-4.863806	-3.074315
1	-3.309583	-3.514772	-2.693799
1	0.121835	-2.326048	-1.672787
1	-1.391361	-1.791256	-2.496846
1	-0.429985	-3.136613	-3.161258
6	-2.844226	-3.700138	1.483251
6	-3.641095	-2.738397	2.376488
6	-1.634851	-4.212789	2.276671
6	-3.741995	-4.862428	1.052918
1	-1.005053	-3.381094	2.626717
1	-1.003456	-4.895489	1.694947
1	-1.994719	-4.759580	3.162782
1	-3.040958	-1.856851	2.654895
1	-3.923004	-3.260304	3.304338
1	-4.555580	-2.385094	1.880841
1	-4.592807	-4.515459	0.446895
1	-4.148891	-5.359333	1.948381
1	-3.186883	-5.616964	0.478120

6	0.232404	1.806085	2.579760
6	-0.926166	1.109981	3.312616
6	0.122134	3.318191	2.799849
6	1.562174	1.274658	3.124520
1	1.698693	0.196967	2.939790
1	1.580887	1.430068	4.215132
1	2.422322	1.813566	2.702246
1	-1.900845	1.371521	2.870198
1	-0.933918	1.429225	4.366719
1	-0.803973	0.016238	3.293938
1	-0.857348	3.697680	2.474961
1	0.906881	3.876566	2.272693
1	0.226144	3.531072	3.875873
6	1.696486	-2.203630	1.330589
8	1.711871	-2.628861	0.155366
8	2.504686	-2.156386	2.234967

Table S18

[P-Int][3]

E : -3560.08529245 Hartree

G : -3559.569569 Hartree

Atomic Number	Coordinates (Angstroms) X	Y	Z
7	-2.420760	0.095898	0.102276

28	-0.936824	-0.941130	0.991513
6	-3.514122	-0.548889	-0.396340
6	-2.365264	1.457901	0.034426
6	-3.656521	-1.899223	-0.456706
15	-2.311133	-2.917608	0.167981
15	0.172851	1.354033	1.010956
6	-1.298595	2.206503	0.415072
1	-4.320636	0.088499	-0.789491
1	-4.567041	-2.320274	-0.886341
1	-3.253830	1.963934	-0.372673
1	-1.343302	3.290437	0.302742
6	1.461443	1.606418	-0.349274
6	-1.488236	-3.619060	-1.384388
15	-0.216456	-2.184630	3.004859
6	2.657898	0.684099	-0.088724
6	1.914840	3.058634	-0.505215
6	0.781028	1.157063	-1.649707
1	2.489567	3.409754	0.362739
1	1.059721	3.734454	-0.658874
1	2.568286	3.140650	-1.389286
1	0.362794	0.143016	-1.556613
1	1.528626	1.138325	-2.458553
1	-0.032957	1.835375	-1.939363
1	3.179959	0.913114	0.848726
1	3.378168	0.786890	-0.916671
1	2.344610	-0.369160	-0.033424
6	-0.556657	-4.781336	-1.033145

6	-0.642032	-2.448981	-1.910328
6	-2.487019	-4.029913	-2.468479
1	0.092459	-4.533158	-0.178637
1	0.090956	-4.998101	-1.898058
1	-1.117439	-5.698791	-0.805042
1	-3.146099	-4.846230	-2.145339
1	-1.934032	-4.378310	-3.356179
1	-3.111987	-3.177993	-2.771947
1	0.183138	-2.220499	-1.215822
1	-1.255131	-1.544754	-2.058636
1	-0.196930	-2.718533	-2.881828
6	-3.154626	-4.243470	1.210286
6	-3.940611	-3.465390	2.276701
6	-2.130838	-5.139538	1.918390
6	-4.115756	-5.113467	0.396270
1	-1.413907	-4.556015	2.513731
1	-1.566308	-5.767847	1.219521
1	-2.668385	-5.810748	2.607661
1	-3.277394	-2.808634	2.861316
1	-4.410247	-4.180145	2.970852
1	-4.728506	-2.844543	1.828238
1	-4.831209	-4.512705	-0.184396
1	-4.695203	-5.751973	1.082962
1	-3.574717	-5.778190	-0.292095
6	0.623973	2.287290	2.594498
6	-0.292207	1.698996	3.676830
6	0.339419	3.789640	2.482700

6	2.085387	2.067414	2.997304
1	2.350905	1.002521	3.044831
1	2.241128	2.502661	3.997784
1	2.779926	2.571982	2.310688
1	-1.351188	1.749519	3.376999
1	-0.172036	2.272967	4.609675
1	-0.041701	0.648497	3.880321
1	-0.733104	3.985843	2.349799
1	0.886590	4.263700	1.656957
1	0.655144	4.278860	3.418407
6	1.371293	-1.658495	2.387397
8	0.744973	-2.259948	1.337622
8	2.457552	-1.185492	2.465936

Table S19

[As-Int]^3

E : -5454.47852803 Hartree

G : -5453.964579 Hartree

Atomic Number	Coordinates (Angstroms) X	Y	Z
7	-2.418195	0.090302	0.115793
28	-0.931432	-0.946137	1.009494
6	-3.511910	-0.554063	-0.381920
6	-2.371184	1.453366	0.057367

6	-3.656138	-1.904183	-0.443929
15	-2.316103	-2.927581	0.182617
15	0.169056	1.361701	1.025832
6	-1.309532	2.208006	0.440382
1	-4.318636	0.083873	-0.773766
1	-4.567910	-2.322838	-0.873394
1	-3.264130	1.955777	-0.344641
1	-1.362954	3.292074	0.333131
6	1.443481	1.601435	-0.352025
6	-1.475297	-3.610437	-1.369297
33	-0.270253	-2.162886	3.178506
6	2.635741	0.669943	-0.104225
6	1.908677	3.048575	-0.519513
6	0.743846	1.155337	-1.643281
1	2.501837	3.396264	0.337358
1	1.058243	3.732863	-0.661318
1	2.548171	3.120636	-1.414604
1	0.313700	0.147323	-1.542004
1	1.482864	1.123777	-2.459598
1	-0.064438	1.842717	-1.927437
1	3.168284	0.892889	0.828818
1	3.349027	0.770046	-0.938592
1	2.316834	-0.381642	-0.048549
6	-0.536075	-4.766594	-1.018326
6	-0.635423	-2.429441	-1.880702
6	-2.460935	-4.021179	-2.465468
1	0.104243	-4.517326	-0.157476

1	0.120425	-4.971836	-1.879324
1	-1.090083	-5.690739	-0.800786
1	-3.109662	-4.851925	-2.159296
1	-1.896440	-4.348520	-3.353941
1	-3.096267	-3.174282	-2.761610
1	0.184547	-2.199762	-1.180417
1	-1.255529	-1.529739	-2.027106
1	-0.182079	-2.688228	-2.851340
6	-3.173808	-4.269575	1.193521
6	-3.987812	-3.510353	2.252417
6	-2.159529	-5.169494	1.909831
6	-4.113619	-5.136363	0.351228
1	-1.460976	-4.591638	2.531757
1	-1.572550	-5.780040	1.213693
1	-2.706139	-5.857189	2.575263
1	-3.345210	-2.848281	2.853782
1	-4.460121	-4.236679	2.932631
1	-4.775699	-2.896325	1.794860
1	-4.815445	-4.533558	-0.243706
1	-4.708735	-5.778994	1.020452
1	-3.555269	-5.797114	-0.326923
6	0.643821	2.310957	2.592442
6	-0.274367	1.759638	3.692131
6	0.386362	3.816797	2.462349
6	2.103652	2.065517	2.986247
1	2.348822	0.995433	3.037800
1	2.276737	2.504139	3.982419

1	2.802481	2.550867	2.290199
1	-1.334264	1.814302	3.396751
1	-0.141990	2.354086	4.610401
1	-0.033719	0.711892	3.920034
1	-0.683309	4.030501	2.332740
1	0.937435	4.271396	1.628857
1	0.714869	4.311759	3.390661
6	1.405612	-1.669731	2.358548
8	0.765391	-2.221626	1.324843
8	2.500695	-1.216174	2.442753

Table S20

[Sb-Int]^3

E : -3459.05666696 Hartree

G : -3458.544504 Hartree

Atomic Number	Coordinates (Angstroms) X	Y	Z
7	-2.155697	0.174080	-0.485657
28	-0.564586	-0.874639	0.170530
6	-3.252970	-0.475125	-0.967359
6	-2.137112	1.532745	-0.423334
6	-3.422785	-1.822805	-0.923308
15	-2.132907	-2.806812	-0.140197
15	0.230841	1.356969	0.893324

6	-1.144526	2.256568	0.161630
1	-4.047343	0.151981	-1.399838
1	-4.339099	-2.261786	-1.320642
1	-2.996100	2.061084	-0.864277
1	-1.197735	3.346417	0.162150
6	1.782656	2.022049	0.062492
6	-1.752708	-4.176873	-1.381136
51	1.653636	-2.372258	1.632547
6	2.972279	1.109273	0.381269
6	2.088756	3.469402	0.452670
6	1.513315	1.939603	-1.448140
1	2.391250	3.560984	1.505712
1	1.227566	4.131435	0.275297
1	2.924236	3.837574	-0.164457
1	1.249572	0.914080	-1.749140
1	2.433404	2.224455	-1.982981
1	0.708257	2.621238	-1.754493
1	3.194144	1.047780	1.455478
1	3.870126	1.497463	-0.125719
1	2.781809	0.097191	-0.002134
6	-0.785645	-5.197962	-0.774844
6	-1.085707	-3.480274	-2.578577
6	-3.012402	-4.884807	-1.894189
1	0.127625	-4.720439	-0.388180
1	-0.472445	-5.902577	-1.561404
1	-1.257611	-5.785419	0.026734
1	-3.531323	-5.461307	-1.120175

1	-2.713101	-5.589717	-2.686397
1	-3.720881	-4.173125	-2.341498
1	-0.118562	-3.028972	-2.326100
1	-1.738493	-2.697016	-2.992853
1	-0.914581	-4.232207	-3.365132
6	-2.910569	-3.448819	1.462804
6	-3.534563	-2.217588	2.136368
6	-1.820987	-4.000609	2.389564
6	-3.996818	-4.504852	1.250242
1	-1.137757	-3.202053	2.713488
1	-1.217216	-4.790293	1.920895
1	-2.291970	-4.424060	3.291466
1	-2.820484	-1.379374	2.194968
1	-3.831649	-2.480417	3.164147
1	-4.421055	-1.864628	1.593073
1	-4.737082	-4.188007	0.500522
1	-4.529520	-4.669004	2.201225
1	-3.571488	-5.469823	0.941547
6	0.144694	1.767993	2.735070
6	-0.976251	0.865017	3.271977
6	-0.236980	3.228920	2.993667
6	1.452816	1.429145	3.453396
1	1.779530	0.397691	3.247902
1	1.300271	1.520406	4.540815
1	2.265916	2.113876	3.173853
1	-1.911197	1.014623	2.707946
1	-1.173759	1.103245	4.329358

1	-0.692952	-0.198562	3.212160
1	-1.219072	3.463640	2.560418
1	0.500869	3.932726	2.586833
1	-0.298004	3.397813	4.081015
6	1.629616	-2.323384	-0.655073
8	0.910608	-1.365344	-1.086552
8	2.221589	-3.184818	-1.247371

Table S21

[Bi-Int]^3

E : -3433.40250448 Hartree

G : -3432.891483 Hartree

Atomic Number	Coordinates (Angstroms) X	Y	Z
7	-2.404809	0.192728	-0.061094
28	-0.824515	-0.851132	0.463468
6	-3.560623	-0.435875	-0.428693
6	-2.292943	1.538117	-0.246578
6	-3.717243	-1.783876	-0.438835
15	-2.260185	-2.729466	0.016239
15	0.137843	1.348503	0.878594
6	-1.185053	2.256954	0.070756
1	-4.392240	0.212862	-0.739690
1	-4.667553	-2.226902	-0.739243

1	-3.163408	2.047405	-0.685215
1	-1.164386	3.333751	-0.097877
6	1.702911	1.624595	-0.120040
6	-1.723026	-3.608487	-1.565161
83	1.350707	-2.110521	3.487556
6	2.846567	0.779311	0.457011
6	2.100364	3.097988	-0.213796
6	1.369360	1.080168	-1.517546
1	2.405728	3.502176	0.762196
1	1.280187	3.716210	-0.609569
1	2.958762	3.201795	-0.897173
1	1.141065	0.003313	-1.470437
1	2.246763	1.205268	-2.171442
1	0.518125	1.608084	-1.971555
1	3.214918	1.164567	1.415817
1	3.690575	0.791630	-0.251252
1	2.524974	-0.264632	0.585795
6	-0.745889	-4.752164	-1.278992
6	-0.989712	-2.521598	-2.368491
6	-2.919614	-4.111853	-2.377805
1	0.113365	-4.426883	-0.671942
1	-0.355382	-5.123390	-2.240506
1	-1.238865	-5.599157	-0.779564
1	-3.511874	-4.861000	-1.835269
1	-2.547522	-4.585515	-3.300601
1	-3.582525	-3.284295	-2.667519
1	-0.050287	-2.246822	-1.865684

1	-1.615071	-1.622971	-2.500740
1	-0.744205	-2.911302	-3.369348
6	-2.793700	-3.910388	1.375102
6	-3.513719	-3.046978	2.420809
6	-1.562818	-4.543038	2.034961
6	-3.746821	-4.994178	0.865003
1	-0.974119	-3.778681	2.563196
1	-0.889323	-5.043585	1.328419
1	-1.894191	-5.281768	2.782312
1	-2.883213	-2.203038	2.747030
1	-3.734150	-3.664491	3.305921
1	-4.457046	-2.637685	2.034296
1	-4.610251	-4.564382	0.334770
1	-4.132316	-5.568678	1.722868
1	-3.239324	-5.701244	0.194252
6	0.206007	2.141735	2.597098
6	-0.911920	1.449728	3.391072
6	-0.096563	3.644053	2.533452
6	1.549962	1.932034	3.297406
1	1.870306	0.880702	3.285281
1	1.453726	2.240338	4.350916
1	2.343601	2.543257	2.844454
1	-1.873220	1.505872	2.855936
1	-1.033670	1.945748	4.367377
1	-0.677566	0.389783	3.573924
1	-1.120689	3.831561	2.185007
1	0.599339	4.183655	1.875695

1	-0.000202	4.068218	3.545909
6	1.260960	-2.766681	1.227259
8	0.778158	-1.897041	0.420535
8	1.669168	-3.870701	0.967362

Table S22

[N-T/S]

E : -3273.47234234 Hartree

Atomic Number	Coordinates (Angstroms) X	Y	Z
7	0.022016	-1.774584	-1.257818
28	0.012911	-0.249427	-0.070686
6	1.206513	-2.277378	-1.709811
6	-1.154804	-2.352271	-1.639542
6	2.413984	-1.759217	-1.365765
15	2.344787	-0.379429	-0.219691
15	-2.314831	-0.403316	-0.234449
6	-2.367634	-1.884164	-1.251651
1	1.149743	-3.141815	-2.386647
1	3.337948	-2.189963	-1.752287
1	-1.082348	-3.234451	-2.290789
1	-3.286221	-2.366103	-1.587899
6	-3.187668	0.943880	-1.209931
6	3.194015	1.068402	-1.067041

7	0.166121	1.194191	1.096301
6	-2.835073	2.319422	-0.631141
6	-4.704282	0.739998	-1.246590
6	-2.631099	0.860471	-2.638917
1	-5.163838	0.889444	-0.259828
1	-4.974596	-0.261807	-1.614244
1	-5.147513	1.478304	-1.933743
1	-1.529124	0.875836	-2.645767
1	-2.985719	1.734645	-3.207082
1	-2.962217	-0.050610	-3.155302
1	-3.064684	2.418413	0.436569
1	-3.399265	3.088540	-1.182596
1	-1.763748	2.539823	-0.755666
6	3.613794	2.158733	-0.077266
6	2.126557	1.645456	-2.011257
6	4.399604	0.589267	-1.882951
1	2.761470	2.523484	0.514405
1	3.993233	3.017611	-0.653835
1	4.422408	1.826445	0.588903
1	5.160629	0.103618	-1.256283
1	4.870283	1.458721	-2.368586
1	4.095197	-0.114136	-2.670501
1	1.329837	2.139450	-1.433421
1	1.694434	0.865939	-2.659860
1	2.590182	2.406091	-2.659086
6	3.197022	-0.941173	1.358381
6	2.559714	-2.290465	1.721412

6	2.921763	0.048084	2.498167
6	4.702255	-1.128223	1.153953
1	1.844928	0.132476	2.701234
1	3.302957	1.055428	2.289636
1	3.417581	-0.318127	3.411641
1	1.461526	-2.218367	1.779405
1	2.927270	-2.603259	2.711383
1	2.811897	-3.070611	0.990356
1	4.916027	-1.800607	0.309107
1	5.137879	-1.576879	2.061348
1	5.213249	-0.171338	0.977974
6	-3.165356	-0.849138	1.382888
6	-2.109647	-1.651468	2.160858
6	-4.397587	-1.730110	1.152862
6	-3.537074	0.398905	2.187529
1	-2.676133	1.068311	2.323763
1	-3.894446	0.081747	3.180722
1	-4.350565	0.964607	1.711670
1	-1.727837	-2.499171	1.569015
1	-2.563214	-2.055029	3.080123
1	-1.266237	-1.005513	2.450537
1	-4.135451	-2.666524	0.640314
1	-5.176104	-1.216308	0.573055
1	-4.831538	-1.991589	2.130961
6	-0.047837	2.560052	1.226271
8	0.611277	3.444025	0.716064
8	-1.082359	2.538071	1.946495

Table S23

[P-T/S]

E : -3560.08318803 Hartree

Atomic Number	Coordinates (Angstroms) X	Y	Z
7	0.070905	-1.736542	-1.314202
28	0.041898	-0.274481	0.034704
6	1.253486	-2.183796	-1.819749
6	-1.099550	-2.315826	-1.713047
6	2.466516	-1.679290	-1.469868
15	2.468417	-0.376956	-0.232484
15	-2.371392	-0.447803	-0.238098
6	-2.324229	-1.900379	-1.301857
1	1.200448	-2.996737	-2.559330
1	3.376916	-2.079348	-1.918613
1	-1.019673	-3.166107	-2.406327
1	-3.220987	-2.403446	-1.666556
6	-3.355839	0.824117	-1.225119
6	3.084806	1.166234	-1.121820
15	0.259185	1.171340	1.900059
6	-3.271731	2.211347	-0.579283
6	-4.819206	0.411592	-1.403143
6	-2.676860	0.892367	-2.601400

1	-5.380644	0.484105	-0.460774
1	-4.914285	-0.612751	-1.794497
1	-5.298654	1.091220	-2.126402
1	-1.595818	1.084935	-2.508760
1	-3.117152	1.723532	-3.174712
1	-2.812263	-0.036479	-3.171786
1	-3.608476	2.221277	0.464562
1	-3.909920	2.902559	-1.153446
1	-2.247539	2.608493	-0.602272
6	3.444520	2.274875	-0.129016
6	1.875901	1.614702	-1.961291
6	4.260060	0.879509	-2.060083
1	2.646059	2.443482	0.611044
1	3.578522	3.218992	-0.680073
1	4.385767	2.062081	0.399379
1	5.148514	0.516444	-1.526643
1	4.539410	1.811665	-2.576959
1	3.983489	0.140642	-2.825753
1	1.070127	2.009041	-1.323102
1	1.490638	0.789579	-2.582453
1	2.182734	2.431369	-2.634188
6	3.619761	-0.986870	1.128504
6	3.084050	-2.373450	1.517693
6	3.552943	-0.081486	2.363850
6	5.068886	-1.109443	0.651351
1	2.539305	-0.042698	2.788074
1	3.874202	0.947612	2.157983

1	4.220883	-0.491255	3.138708
1	2.018638	-2.328965	1.796729
1	3.646068	-2.744550	2.389256
1	3.190960	-3.096638	0.697927
1	5.148959	-1.689919	-0.279901
1	5.661973	-1.629116	1.421224
1	5.526925	-0.122813	0.492243
6	-3.255187	-1.025667	1.325723
6	-2.187553	-1.815149	2.100814
6	-4.429168	-1.965630	1.034173
6	-3.723021	0.164247	2.167866
1	-2.928257	0.911733	2.305582
1	-4.033115	-0.200073	3.160585
1	-4.591639	0.663206	1.713421
1	-1.723416	-2.589557	1.467975
1	-2.655101	-2.317259	2.962605
1	-1.397595	-1.152395	2.483809
1	-4.093371	-2.873843	0.514314
1	-5.214323	-1.487733	0.434433
1	-4.881717	-2.273045	1.991049
6	-0.212405	2.674757	1.016411
8	-0.017862	3.546617	0.226011
8	-1.224175	2.310225	1.754357

Table S24

[As-T/S]

E : -5454.47416998 Hartree

Atomic	Coordinates (Angstroms)		
Number	X	Y	Z
7	-0.025621	-2.217470	0.701572
28	-0.010078	-0.368901	-0.024132
6	-1.202936	-2.821472	1.018153
6	1.144410	-2.909313	0.834545
6	-2.419025	-2.231600	0.875706
15	-2.433556	-0.577495	0.179320
15	2.394993	-0.633934	0.125804
6	2.364824	-2.375772	0.577567
1	-1.143925	-3.845651	1.415398
1	-3.326669	-2.769702	1.151671
1	1.065623	-3.954994	1.166626
1	3.266102	-2.973583	0.719300
6	3.399515	0.193354	1.493860
6	-3.104324	0.535014	1.548062
33	-0.307197	1.726642	-1.265356
6	3.201849	1.712205	1.465772
6	4.891026	-0.139972	1.414256
6	2.835075	-0.344288	2.817221
1	5.371077	0.340243	0.550268
1	5.072994	-1.223966	1.362058
1	5.389081	0.241488	2.320224
1	1.738282	-0.248261	2.861698

1	3.261707	0.243193	3.645324
1	3.089092	-1.401794	2.970428
1	3.431585	2.159945	0.491090
1	3.863956	2.165912	2.221039
1	2.169318	1.990258	1.719123
6	-3.502584	1.913609	1.015657
6	-1.919824	0.692821	2.516325
6	-4.277147	-0.104099	2.297790
1	-2.692731	2.385389	0.436464
1	-3.716315	2.575535	1.869915
1	-4.410492	1.867343	0.396359
1	-5.148305	-0.272423	1.651679
1	-4.590674	0.571712	3.109704
1	-3.985720	-1.062138	2.750930
1	-1.113086	1.295825	2.071673
1	-1.522930	-0.286589	2.828520
1	-2.259482	1.223215	3.420181
6	-3.564594	-0.689245	-1.327305
6	-3.030994	-1.865914	-2.157838
6	-3.475101	0.581222	-2.180680
6	-5.021319	-0.954177	-0.938582
1	-2.477767	0.697619	-2.629203
1	-3.705872	1.495975	-1.619185
1	-4.196141	0.507434	-3.011090
1	-1.956941	-1.749821	-2.377382
1	-3.570314	-1.902439	-3.117711
1	-3.168982	-2.824931	-1.641254

1	-5.116163	-1.815430	-0.260192
1	-5.605136	-1.176619	-1.846539
1	-5.478450	-0.076764	-0.459746
6	3.249474	-0.579444	-1.555900
6	2.199392	-1.116561	-2.541836
6	4.485567	-1.481790	-1.637243
6	3.613140	0.860665	-1.928248
1	2.764627	1.548365	-1.798953
1	3.929677	0.893765	-2.983448
1	4.449517	1.236978	-1.321282
1	1.834440	-2.110358	-2.236350
1	2.650820	-1.211804	-3.542327
1	1.339727	-0.435408	-2.621456
1	4.237439	-2.527772	-1.407691
1	5.291452	-1.158249	-0.968429
1	4.879723	-1.450902	-2.665984
6	0.272713	2.874087	0.209460
8	0.039652	3.340876	1.280729
8	1.264411	2.857522	-0.602892

Table S25

[Sb-T/S]

E : -3459.02863787 Hartree

Atomic	Coordinates (Angstroms)		
Number	X	Y	Z

7	-0.009389	2.459957	-0.386075
28	0.004817	0.477375	-0.280144
6	1.150777	3.164078	-0.307362
6	-1.185936	3.119218	-0.596978
6	2.369268	2.589685	-0.124057
15	2.384614	0.798547	-0.036594
15	-2.389368	0.756155	-0.152317
6	-2.395181	2.505363	-0.585355
1	1.074268	4.258320	-0.389326
1	3.268995	3.203395	-0.061434
1	-1.121530	4.202451	-0.777460
1	-3.305628	3.084209	-0.742402
6	-3.139262	0.712394	1.583123
6	2.904195	0.377541	1.729313
51	0.312059	-2.109764	-0.670889
6	-3.002643	-0.675124	2.217436
6	-4.604374	1.154207	1.599089
6	-2.303413	1.696147	2.415682
1	-5.262992	0.399886	1.145610
1	-4.754154	2.114180	1.081927
1	-4.924322	1.283800	2.645745
1	-1.228778	1.463586	2.348285
1	-2.601218	1.606850	3.472212
1	-2.451091	2.735900	2.094866
1	-3.427332	-1.480712	1.607399
1	-3.525953	-0.665522	3.187180

1	-1.952159	-0.927930	2.415694
6	3.337961	-1.083497	1.870960
6	1.628763	0.599409	2.557841
6	3.999416	1.315966	2.244400
1	2.562699	-1.781771	1.517211
1	3.495563	-1.301977	2.938935
1	4.283464	-1.287399	1.347577
1	4.933166	1.229349	1.673219
1	4.224472	1.059833	3.291842
1	3.668127	2.363742	2.218443
1	0.860256	-0.150397	2.312394
1	1.224583	1.613242	2.406159
1	1.862195	0.482441	3.627971
6	3.643596	0.250476	-1.325716
6	3.257487	0.979752	-2.620290
6	3.534158	-1.258314	-1.573811
6	5.079012	0.621798	-0.944186
1	2.589117	-1.504200	-2.085140
1	3.610490	-1.859537	-0.657520
1	4.341512	-1.580126	-2.250884
1	2.197978	0.814980	-2.874531
1	3.871014	0.592719	-3.449010
1	3.420954	2.062245	-2.539207
1	5.171293	1.690839	-0.700658
1	5.744541	0.414740	-1.798072
1	5.444503	0.034083	-0.090571
6	-3.486604	-0.062495	-1.446791

6	-2.617117	-0.146144	-2.710569
6	-4.726417	0.774595	-1.781557
6	-3.904205	-1.468596	-1.005154
1	-3.056004	-2.068819	-0.642081
1	-4.356777	-1.993365	-1.861700
1	-4.659729	-1.429048	-0.206692
1	-2.215495	0.843742	-2.980540
1	-3.229553	-0.507903	-3.551812
1	-1.774178	-0.838452	-2.577871
1	-4.446597	1.749614	-2.204276
1	-5.373855	0.941860	-0.911918
1	-5.317678	0.238913	-2.541906
6	-0.302561	-2.576648	1.417690
8	0.066206	-2.533601	2.548209
8	-1.341023	-2.835822	0.761248

Table S26

[Bi-T/S]

E : -3433.36653288 Hartree

Atomic	Coordinates (Angstroms)		
Number	X	Y	Z
7	0.122112	2.756053	-0.162362
28	0.045929	0.800699	-0.158371
6	1.323642	3.393208	-0.158962

6	-1.020586	3.492188	-0.251458
6	2.505271	2.732053	-0.064286
15	2.347110	0.950246	0.069241
15	-2.258663	1.139933	-0.089413
6	-2.254057	2.930810	-0.252911
1	1.306297	4.490378	-0.229463
1	3.455668	3.266309	-0.055440
1	-0.905064	4.583482	-0.319648
1	-3.152321	3.546270	-0.301261
6	-3.031518	0.834466	1.609279
6	2.934471	0.551165	1.825952
83	0.300558	-1.772042	-0.551636
6	-2.855509	-0.627065	2.025206
6	-4.508311	1.226937	1.677302
6	-2.226919	1.707463	2.584141
1	-5.141154	0.548560	1.087541
1	-4.672978	2.256892	1.326977
1	-4.848741	1.171158	2.724122
1	-1.145281	1.525489	2.484828
1	-2.521585	1.458662	3.615902
1	-2.408241	2.777039	2.414952
1	-3.382845	-1.315724	1.352023
1	-3.257137	-0.766899	3.041875
1	-1.784751	-0.889426	2.041468
6	3.328220	-0.916567	2.007681
6	1.713706	0.851489	2.708807
6	4.089900	1.463510	2.252313

1	2.524869	-1.594008	1.677673
1	3.504306	-1.101451	3.079616
1	4.259464	-1.164974	1.477828
1	4.980464	1.341506	1.620905
1	4.377455	1.209926	3.285307
1	3.791039	2.520453	2.234641
1	0.904739	0.132711	2.504947
1	1.338272	1.874282	2.540669
1	1.998745	0.762446	3.769472
6	3.487950	0.238584	-1.239720
6	3.116787	0.931300	-2.558919
6	3.201200	-1.257952	-1.381390
6	4.968743	0.479265	-0.937764
1	2.210500	-1.404630	-1.868775
1	3.281483	-1.814073	-0.439298
1	3.900508	-1.716776	-2.097551
1	2.039933	0.837218	-2.770691
1	3.670613	0.453235	-3.382559
1	3.371138	1.999449	-2.538807
1	5.180365	1.547179	-0.778201
1	5.570695	0.145794	-1.798354
1	5.309377	-0.081252	-0.056554
6	-3.339154	0.554394	-1.519622
6	-2.437300	0.655129	-2.759570
6	-4.562288	1.453351	-1.733827
6	-3.780967	-0.896476	-1.330325
1	-2.925835	-1.560556	-1.144482

1	-4.274990	-1.244307	-2.251457
1	-4.503039	-1.003998	-0.507085
1	-2.039232	1.675593	-2.877504
1	-3.032023	0.415191	-3.655736
1	-1.593764	-0.047430	-2.696469
1	-4.263647	2.482685	-1.975086
1	-5.235471	1.476166	-0.867218
1	-5.135531	1.065068	-2.591079
6	-1.455842	-3.377221	1.343291
8	-1.196821	-3.800662	2.380539
8	-1.847016	-2.994714	0.308205

Table S27

[N-Prod][1]

E : -3273.50118826 Hartree

G : -3272.978215 Hartree

Atomic Number	Coordinates (Angstroms) X	Y	Z
7	-2.459127	0.174230	-0.190070
28	-0.950072	-0.781632	0.558080
6	-3.518751	-0.515035	-0.704866
6	-2.506374	1.534934	-0.124251
6	-3.578389	-1.870444	-0.737329
15	-2.170912	-2.707371	0.005038

15	-0.038779	1.348317	0.839617
6	-1.490939	2.290409	0.370619
1	-4.349621	0.084705	-1.103278
1	-4.445118	-2.378662	-1.160321
1	-3.422313	2.015695	-0.496670
1	-1.556373	3.378447	0.387497
6	1.307261	1.802762	-0.395200
6	-1.454535	-3.800070	-1.358981
7	0.396599	-1.673553	1.552579
6	2.430711	0.762472	-0.332970
6	1.855737	3.214852	-0.181401
6	0.649437	1.720089	-1.780730
1	2.425033	3.299231	0.754647
1	1.051153	3.966077	-0.173434
1	2.539378	3.465418	-1.008680
1	0.132153	0.757891	-1.926563
1	1.431906	1.805463	-2.551048
1	-0.082957	2.523804	-1.933483
1	2.894984	0.672236	0.656473
1	3.214884	1.036259	-1.057032
1	2.055205	-0.234823	-0.607331
6	-0.695892	-5.019256	-0.826868
6	-0.470935	-2.879408	-2.097548
6	-2.550917	-4.261338	-2.325437
1	0.074725	-4.734944	-0.100013
1	-0.199609	-5.514188	-1.677303
1	-1.372640	-5.755355	-0.370154

1	-3.332368	-4.843832	-1.815241
1	-2.099368	-4.907011	-3.095700
1	-3.027301	-3.410226	-2.830530
1	0.375993	-2.621776	-1.442354
1	-0.965441	-1.952797	-2.432449
1	-0.076023	-3.394659	-2.987822
6	-2.857876	-3.677934	1.460172
6	-3.574179	-2.638555	2.335532
6	-1.719928	-4.293256	2.284146
6	-3.856791	-4.747804	1.013948
1	-0.989876	-3.528266	2.584677
1	-1.180941	-5.078891	1.741731
1	-2.149971	-4.744649	3.192938
1	-2.880400	-1.845914	2.658651
1	-3.959534	-3.136896	3.238936
1	-4.416605	-2.168849	1.809214
1	-4.673074	-4.315523	0.415290
1	-4.306930	-5.219936	1.902329
1	-3.374179	-5.541647	0.426448
6	0.376380	1.819992	2.610093
6	-0.640014	1.046038	3.465257
6	0.197321	3.321254	2.856696
6	1.793082	1.379659	2.989822
1	1.982897	0.319832	2.763291
1	1.927407	1.521035	4.074171
1	2.560905	1.981216	2.482475
1	-1.673742	1.254882	3.145434

1	-0.540177	1.355847	4.517703
1	-0.460799	-0.037850	3.405007
1	-0.839239	3.634569	2.667984
1	0.866715	3.931545	2.235756
1	0.428165	3.539677	3.911777
6	1.675549	-2.008379	1.466944
8	2.813683	-1.697846	1.628839
8	0.983431	-3.088546	1.098631

Table S28

[P-Prod][1]

E : -3560.08983190 Hartree

G : -3559.571165 Hartree

Atomic Number	Coordinates (Angstroms) X	Y	Z
7	-2.394506	0.180608	-0.218099
28	-0.888673	-0.789682	0.564193
6	-3.520301	-0.495801	-0.580723
6	-2.354354	1.534039	-0.319011
6	-3.625990	-1.844696	-0.492440
15	-2.154214	-2.703146	0.091089
15	0.055186	1.342703	0.825149
6	-1.295786	2.275667	0.101558
1	-4.363107	0.102612	-0.956436

1	-4.540030	-2.352013	-0.801117
1	-3.232559	2.029419	-0.758700
1	-1.294319	3.360794	-0.001157
6	1.578525	1.762497	-0.197403
6	-1.641243	-3.759669	-1.397527
15	0.842699	-1.718260	1.706842
6	2.799084	0.925019	0.204542
6	1.913610	3.254225	-0.115138
6	1.203748	1.390630	-1.640042
1	2.304390	3.529232	0.875348
1	1.042173	3.887366	-0.339670
1	2.696574	3.487906	-0.854278
1	0.935017	0.324694	-1.715813
1	2.077021	1.559217	-2.289331
1	0.364344	1.992374	-2.014826
1	3.071933	1.036028	1.261112
1	3.659896	1.263142	-0.394779
1	2.656116	-0.140316	-0.019981
6	-0.831027	-5.003816	-1.024255
6	-0.779737	-2.820945	-2.256954
6	-2.868671	-4.185459	-2.213930
1	0.032311	-4.762267	-0.392130
1	-0.457348	-5.462922	-1.953596
1	-1.451337	-5.755984	-0.515190
1	-3.589017	-4.764332	-1.618321
1	-2.531001	-4.824786	-3.045181
1	-3.388609	-3.318530	-2.642423

1	0.194117	-2.616746	-1.787751
1	-1.293792	-1.863548	-2.443368
1	-0.585713	-3.295572	-3.232176
6	-2.732549	-3.728716	1.557738
6	-3.350303	-2.722822	2.540021
6	-1.557918	-4.425208	2.253690
6	-3.790849	-4.756898	1.148171
1	-0.837183	-3.695850	2.650226
1	-1.011721	-5.109499	1.593939
1	-1.948234	-5.006060	3.104900
1	-2.620080	-1.949233	2.828221
1	-3.656565	-3.254309	3.454862
1	-4.230642	-2.222632	2.113054
1	-4.634539	-4.287001	0.621017
1	-4.191185	-5.243917	2.052029
1	-3.370439	-5.544377	0.507118
6	0.157502	1.953755	2.610272
6	-0.960515	1.194830	3.343313
6	-0.121749	3.457071	2.712832
6	1.503134	1.627254	3.262939
1	1.774990	0.565907	3.151124
1	1.434327	1.839713	4.341916
1	2.315642	2.245385	2.855377
1	-1.926239	1.300768	2.823346
1	-1.073870	1.603029	4.360326
1	-0.725276	0.123258	3.434414
1	-1.134583	3.697056	2.361624

1	0.597362	4.058282	2.140351
1	-0.048197	3.761799	3.769223
6	1.764749	-2.469927	0.348569
8	2.472750	-2.429888	-0.608920
8	1.088453	-3.409739	0.953606

Table S29

[As-Prod][1]

E : -5454.48023119 Hartree

G : -5453.962878 Hartree

Atomic	Coordinates (Angstroms)		
Number	X	Y	Z
7	-2.405756	0.179625	-0.180931
28	-0.914445	-0.780998	0.638482
6	-3.529985	-0.500847	-0.544026
6	-2.365626	1.530847	-0.298621
6	-3.632401	-1.848533	-0.450255
15	-2.155527	-2.707212	0.124259
15	0.037247	1.355306	0.858505
6	-1.310116	2.278890	0.118892
1	-4.372240	0.094492	-0.925484
1	-4.543326	-2.358896	-0.763170
1	-3.240562	2.019930	-0.751597
1	-1.306074	3.362158	-0.000935

6	1.552076	1.710041	-0.202778
6	-1.614944	-3.699466	-1.400758
33	0.856347	-1.719039	1.905777
6	2.760172	0.857331	0.205166
6	1.920273	3.195899	-0.175403
6	1.143937	1.301891	-1.626427
1	2.335236	3.494997	0.798051
1	1.057806	3.839502	-0.405043
1	2.693767	3.388379	-0.936032
1	0.836403	0.244299	-1.662358
1	2.013148	1.417206	-2.292559
1	0.320461	1.919535	-2.010392
1	3.047146	0.986168	1.256486
1	3.621844	1.163787	-0.409630
1	2.593376	-0.210278	0.006910
6	-0.813139	-4.960528	-1.068975
6	-0.739116	-2.723941	-2.203059
6	-2.827025	-4.088914	-2.257340
1	0.038608	-4.753323	-0.409319
1	-0.422278	-5.377369	-2.011084
1	-1.445309	-5.733433	-0.607649
1	-3.558458	-4.691471	-1.699964
1	-2.473437	-4.693588	-3.107657
1	-3.339023	-3.204596	-2.658750
1	0.225901	-2.539570	-1.707646
1	-1.251767	-1.759888	-2.357404
1	-0.526452	-3.155950	-3.194109

6	-2.752914	-3.794521	1.538480
6	-3.376921	-2.829387	2.557077
6	-1.594817	-4.533390	2.217556
6	-3.811427	-4.796858	1.068310
1	-0.872004	-3.831652	2.656735
1	-1.045890	-5.192617	1.535130
1	-2.003882	-5.147983	3.035627
1	-2.642535	-2.080105	2.894650
1	-3.705607	-3.400347	3.439764
1	-4.243997	-2.298613	2.139715
1	-4.649112	-4.299977	0.556931
1	-4.221562	-5.325763	1.943737
1	-3.386650	-5.553794	0.394088
6	0.176009	2.039350	2.614238
6	-0.946735	1.338112	3.395511
6	-0.073280	3.551349	2.655344
6	1.525521	1.720493	3.262267
1	1.781452	0.650460	3.204325
1	1.478923	1.987609	4.330112
1	2.343366	2.299731	2.810907
1	-1.917525	1.448614	2.886544
1	-1.032361	1.789141	4.396937
1	-0.741281	0.264523	3.525747
1	-1.086674	3.795424	2.308981
1	0.648309	4.113804	2.047882
1	0.021147	3.899295	3.696553
6	1.791071	-2.620434	0.451016

8	2.496163	-2.551003	-0.506637
8	1.108153	-3.542188	1.032686

Table S30

[Sb-Prod][1]

E : -3459.03210495 Hartree

G : -3458.516627 Hartree

Atomic Number	Coordinates (Angstroms) X	Y	Z
7	-2.441098	0.182826	-0.120227
28	-0.968290	-0.768118	0.748205
6	-3.553690	-0.505118	-0.506077
6	-2.408029	1.533083	-0.239576
6	-3.645140	-1.853499	-0.421622
15	-2.168836	-2.705982	0.165560
15	-0.004348	1.369477	0.914123
6	-1.359157	2.287982	0.183439
1	-4.393901	0.084873	-0.900007
1	-4.544532	-2.370718	-0.756241
1	-3.281075	2.016593	-0.702153
1	-1.356468	3.370133	0.054999
6	1.474785	1.640024	-0.225698
6	-1.553855	-3.612581	-1.387119
51	0.868471	-1.749139	2.237357

6	2.670941	0.758066	0.155209
6	1.893374	3.111816	-0.269616
6	0.988626	1.203112	-1.615809
1	2.363854	3.433065	0.670911
1	1.041766	3.774874	-0.484201
1	2.635407	3.249776	-1.072258
1	0.615142	0.166881	-1.598188
1	1.836344	1.240054	-2.317597
1	0.188454	1.854427	-1.992962
1	3.005119	0.902710	1.191358
1	3.517548	1.020824	-0.499348
1	2.464309	-0.309763	-0.004862
6	-0.745126	-4.877242	-1.088261
6	-0.662754	-2.584745	-2.102110
6	-2.723742	-3.975217	-2.311086
1	0.075820	-4.699402	-0.382150
1	-0.307571	-5.236792	-2.033430
1	-1.383363	-5.683197	-0.697276
1	-3.462207	-4.622945	-1.817321
1	-2.325113	-4.523830	-3.179347
1	-3.239782	-3.079836	-2.681540
1	0.271075	-2.401395	-1.548547
1	-1.191445	-1.627149	-2.242320
1	-0.388642	-2.969074	-3.097710
6	-2.801801	-3.877665	1.494715
6	-3.486184	-2.981245	2.536888
6	-1.660648	-4.636279	2.179892

6	-3.820661	-4.871097	0.927044
1	-0.985397	-3.950465	2.711233
1	-1.054093	-5.228215	1.485039
1	-2.093195	-5.318157	2.929588
1	-2.784502	-2.232163	2.938285
1	-3.831803	-3.604621	3.376644
1	-4.351247	-2.451169	2.114539
1	-4.646022	-4.362892	0.407199
1	-4.256832	-5.451854	1.755714
1	-3.353998	-5.584544	0.233580
6	0.225688	2.149276	2.620386
6	-0.883109	1.536445	3.490217
6	0.022378	3.668915	2.583074
6	1.593427	1.833435	3.229796
1	1.821494	0.754761	3.244228
1	1.606435	2.175421	4.276844
1	2.408080	2.347396	2.700897
1	-1.870640	1.654933	3.017127
1	-0.906907	2.046594	4.466210
1	-0.716137	0.464215	3.674722
1	-0.998579	3.923924	2.268830
1	0.731130	4.173787	1.913712
1	0.172883	4.072110	3.597350
6	1.880606	-2.858185	0.622904
8	2.577556	-2.755996	-0.336029
8	1.190893	-3.749800	1.196252

Table S31

[Bi-Prod][1]

E : -3433.36387309 Hartree

G : -3432.850303 Hartree

Atomic Number	Coordinates (Angstroms) X	Y	Z
7	-2.450098	0.185245	-0.116311
28	-0.985303	-0.760574	0.774175
6	-3.553315	-0.508094	-0.518680
6	-2.428686	1.536640	-0.218842
6	-3.636542	-1.857485	-0.442665
15	-2.164168	-2.705663	0.161611
15	-0.028066	1.380117	0.941014
6	-1.389436	2.296546	0.220205
1	-4.393220	0.078272	-0.918729
1	-4.528417	-2.378389	-0.791506
1	-3.302651	2.017813	-0.682270
1	-1.395181	3.379792	0.101380
6	1.434863	1.630702	-0.227545
6	-1.523624	-3.598763	-1.389115
83	0.864773	-1.697663	2.383025
6	2.637552	0.753637	0.142118
6	1.857174	3.099934	-0.304852
6	0.920601	1.171731	-1.600560

1	2.356653	3.431888	0.616980
1	1.002587	3.763865	-0.504372
1	2.576785	3.223172	-1.130007
1	0.545457	0.136945	-1.557749
1	1.754338	1.195510	-2.319452
1	0.114138	1.818180	-1.972692
1	3.011821	0.936701	1.158295
1	3.460171	0.984395	-0.553919
1	2.417116	-0.318201	0.037904
6	-0.721441	-4.866242	-1.085653
6	-0.619085	-2.566250	-2.079577
6	-2.675772	-3.954263	-2.337578
1	0.080439	-4.700431	-0.355386
1	-0.258274	-5.214605	-2.022792
1	-1.368328	-5.677281	-0.720566
1	-3.423325	-4.605367	-1.862173
1	-2.261766	-4.496177	-3.202941
1	-3.184972	-3.055793	-2.709946
1	0.300851	-2.381904	-1.503014
1	-1.146712	-1.608989	-2.225553
1	-0.321882	-2.944291	-3.071000
6	-2.813405	-3.893380	1.468862
6	-3.511219	-3.008145	2.511729
6	-1.679403	-4.655890	2.161074
6	-3.823555	-4.882323	0.878647
1	-1.002688	-3.970939	2.692153
1	-1.075035	-5.256162	1.471459

1	-2.118347	-5.332072	2.912250
1	-2.812694	-2.267031	2.932547
1	-3.871280	-3.641486	3.337886
1	-4.368724	-2.470970	2.082779
1	-4.644049	-4.369824	0.355332
1	-4.268043	-5.472981	1.695827
1	-3.348294	-5.587571	0.182600
6	0.232153	2.178969	2.633003
6	-0.884218	1.611613	3.523411
6	0.070973	3.703239	2.579140
6	1.597346	1.833155	3.230453
1	1.807204	0.748930	3.243105
1	1.630915	2.171874	4.277944
1	2.419186	2.325109	2.692018
1	-1.872357	1.753183	3.058537
1	-0.881424	2.136177	4.492014
1	-0.750575	0.536779	3.720032
1	-0.945121	3.981676	2.268980
1	0.788112	4.181741	1.900026
1	0.237881	4.113980	3.587851
6	1.930000	-3.004155	0.677971
8	2.625902	-2.778037	-0.253340
8	1.287390	-3.878406	1.257830
